# Brain metastatic cancer cells release microRNA-181c-containing extracellular vesicles capable of destructing blood–brain barrier

**DOI:** 10.1038/ncomms7716

**Published:** 2015-04-01

**Authors:** Naoomi Tominaga, Nobuyoshi Kosaka, Makiko Ono, Takeshi Katsuda, Yusuke Yoshioka, Kenji Tamura, Jan Lötvall, Hitoshi Nakagama, Takahiro Ochiya

**Affiliations:** 1 Division of Molecular and Cellular Medicine, National Cancer Center Research Institute, 5-1-1 Tsukiji, Chuo-ku, Tokyo 104-0045, Japan; 2 Graduate School of Medicine, The University of Tokyo, 7-3-1 Hongo, Bunkyou-ku, Tokyo 113-0033, Japan; 3 Research Fellow of the Japan Society for the Promotion of Science (JSPS), Chiyoda-Ku, Tokyo 102-0083, Japan; 4 Department of Zoology, University of Oxford, The Tinbergen Building, South Parks Road, Oxford OX1 3PS, United Kingdom; 5 The Japan Society for the Promotion of Science (JSPS) Postdoctoral Fellow for Research Abroad, Chiyoda-Ku, Tokyo 102-0083, Japan; 6 Division of Breast and Medical Oncology, National Cancer Center Research Institute, 5-1-1 Tsukiji, Chuo-ku, Tokyo 104-0045, Japan; 7 Department of Internal Medicine and Department of Respiratory Medicine and Allergology, The Sahlgrenska Academy, University of Göteborg, Box 424, SE-405 30 Gothenburg, Sweden; 8 Division of Cancer Development System, National Cancer Center Research Institute, 5-1-1 Tsukiji, Chuo-ku, Tokyo 104-0045, Japan

## Abstract

Brain metastasis is an important cause of mortality in breast cancer patients. A key event during brain metastasis is the migration of cancer cells through blood–brain barrier (BBB). However, the molecular mechanism behind the passage through this natural barrier remains unclear. Here we show that cancer-derived extracellular vesicles (EVs), mediators of cell–cell communication via delivery of proteins and microRNAs (miRNAs), trigger the breakdown of BBB. Importantly, miR-181c promotes the destruction of BBB through the abnormal localization of actin via the downregulation of its target gene, *PDPK1*. PDPK1 degradation by miR-181c leads to the downregulation of phosphorylated cofilin and the resultant activated cofilin-induced modulation of actin dynamics. Furthermore, we demonstrate that systemic injection of brain metastatic cancer cell-derived EVs promoted brain metastasis of breast cancer cell lines and are preferentially incorporated into the brain *in vivo*. Taken together, these results indicate a novel mechanism of brain metastasis mediated by EVs that triggers the destruction of BBB.

## Abstract (web summary)

A key event during metastasis to the brain is the migration of cancer cells through the blood–brain barrier (BBB). Here the authors show that cancer-cell-derived extracellular vesicles promote metastasis by promoting BBB breaching.

## Graphical abstract

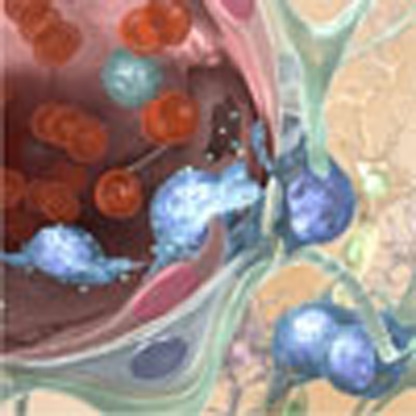

Brain metastasis is associated with a particularly poor prognosis for cancer patients. Therefore, novel insights into the brain metastatic process are needed. A key event during brain metastasis is the migration of cancer cells through blood–brain barrier (BBB)^1^,^2^, which consists of the endothelium and surrounding cells^3^,^4^. BBB limits the passive diffusion of molecules. One of the key features of brain metastasis is the destruction of BBB^5^. Tumour cells recognize and bind to components of the vascular membrane, thereby initiating extravasation, invasion of cancer cells through BBB and the beginning of new growth at secondary organ sites^6^,^7^. This process suggests that the intact endothelium can serve as a defensive barrier against the extravasation of tumour cells. Although these hypotheses are accepted, the exact molecular mechanisms that trigger brain metastasis are poorly understood. Humoral factors have been proposed to mediate the interaction between cancer cells and BBB-constructed cells, resulting in the destruction of BBB structure. However, the actions of these molecules do not fully explain the destruction of BBB during brain metastasis, given the complexity of the interactions among cells that comprise BBB^8^. Extracellular vesicles (EVs), including exosomes, are known to regulate multiple aspects of malignancy in cancer cells^9^,^10^,^11^. EVs are known to mediate cell–cell communication via the delivery of their contents, including proteins, mRNAs and microRNAs (miRNAs)^12^. Although the contribution of EVs to cancer metastasis is evident, little is known regarding the roles of EVs in brain metastasis.

In the present study, we report that cancer-derived EVs break down BBB through the change in actin dynamics *in vitro*, and that they promote brain metastasis *in vivo*. Furthermore, *in vitro* study, miR-181c promotes the destruction of BBB through delocalization of actin fibre via the downregulation of 3-phosphoinositide-dependent protein kinase-1 (PDPK1). PDPK1 degradation by miR-181c leads to the downregulation of phosphorylated cofilin and the resultant activated cofilin-induced modulation of actin dynamics. In addition, our clinical data suggest that cancer-derived EVs cause brain metastases in cancer patients.

## Results

### Establishment of brain metastasis breast cancer cell lines

**Figure 1:**
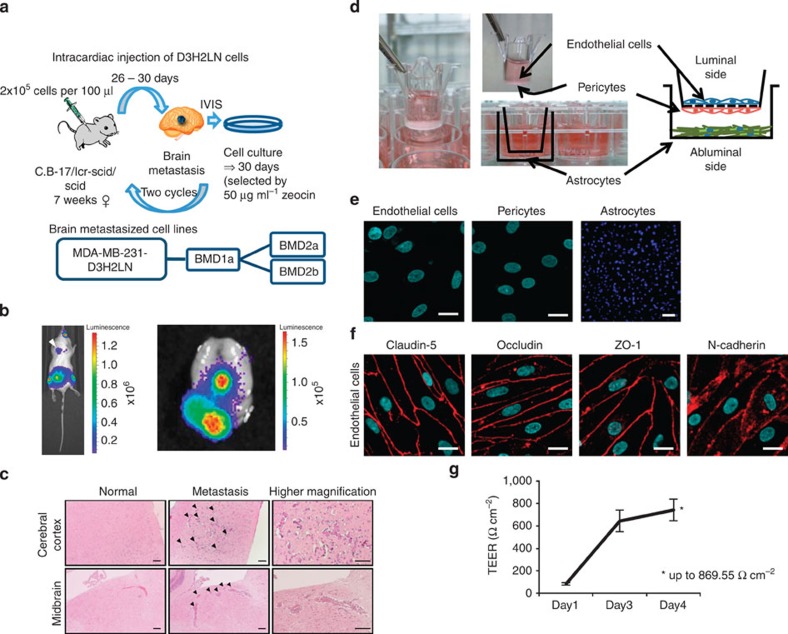
Establishment of brain metastasis breast cancer cell lines and BBB *in vitro* model. (**a**) Schematic representation of the protocol for the *in vivo*-selected brain metastatic derivatives. MDA-MB-231-luc-D3H2LN breast cancer cell lines (2 × 10^5^ cells) were injected intracardially into C.B-17 Icr-scid scid mice. After 26–30 days, the brain metastasis of cancer cells was monitored by *in vivo* imaging system (IVIS). The brain-metastasized cancer cells were recovered and cultured for ~30 days in a culture medium containing 50 μg ml^−1^ Zeocin. This selection was performed twice, and we named the established cell lines BMD2a and BMD2b. (**b**) Bioluminescence image of a mouse with a BMD2a brain metastasis (left). Right image represents the bioluminescence image of a mouse brain with cancer cell metastasis. (**c**) Representative image of HE-stained sections from a mouse brain cerebral cortex and midbrain. Left upper and lower panels show the mouse cerebral cortex and midbrain, respectively, without metastasis of cancer cells. Middle upper and lower panels show the mouse cerebral cortex and midbrain, respectively, with metastasis of cancer cells. Arrow-head represent metastatic cancer cells. Right upper and lower panels show higher magnification. Scale bar, 100 μm. (**d**) The schematic representation of the *in vitro* model of BBB constructed from primary cultures of monkey brain capillary endothelial cells, brain pericytes and astrocytes. (**e**) Representative pictures of endothelial cells, pericytes and astrocytes are shown. Endothelial cells and pericytes were visualized using a confocal microscope. Astrocytes were visualized using a fluorescence microscope. Scale bar, 20 μm. Scale bar in the panel of astrocytes represents 100 μm. (**f**) Immunofluorescence of tight junction proteins (Claudin-5, Occludin and ZO-1) and N-cadherin (red). Scale bar, 20 μm. (**g**) The transition of TEER after thawing until the start of the experiment. After thawing the BBB *in vitro* model, the value of TEER increased to a maximum of 869.55 Ω cm^−2^ (*mean maximum TEER.). Error bars represent s.d., *n*=12. Data are representative of at least three independent experiments each. (Fig. e,f,g).

To identify the influence of EVs on BBB during breast cancer metastasis, we employed MDA-MB-231-luc-D3H2LN cells (D3H2LN), which are human mammary tumour cells with a high tumorigenic and metastatic capacity^13^,^14^ to produce a new brain high-metastatic cell line. D3H2LN cells were injected into immunodeficient female mice by intracardiac (i.c.) injections to isolate populations of cells that colonized in the brain (Fig. 1a). Brain metastasis was monitored by *in vivo* imaging using intraperitoneal luciferin injections (Fig. 1b). Cancer cell colonization of the brain tissue was also confirmed by hematoxylin and eosin (HE) staining (Fig. 1c). After tumour dissociation and expansion in culture, the resulting cell populations (brain metastatic derivative 1a, BMD1a) were subjected to a second round of *in vivo* selection, yielding brain metastatic derivative cell populations 2a and 2b (BMD2a and BMD2b), which showed significant increases in brain metastatic activity over the original cell line. When injected into the left ventricle, BMD1a cells metastasized to the brain in 60% (3 out of 5) of the mice, whereas D3H2LN cells yielded 1 brain metastasis out of 15 injected mice (6.7%).

### BBB *in vitro* model

To determine how the EVs from breast cancer cells that metastasize to the brain affect BBB, an *in vitro* BBB culture system that enables us to study the molecular and cellular effects of the EVs is essential. To this end, recent studies have employed monolayer cell culture systems^15^. However, BBB consists of three different types of cell, and these cells cooperate with each other to maintain the structure of BBB. Therefore, we employed a new *in vitro* BBB model system that consists of primary cultures of brain capillary endothelial cells, brain pericytes and astrocytes (Fig. 1d). As shown in Fig. 1e, brain capillary endothelial cells, brain pericytes and astrocytes were assessed using Hoechst 33342 staining. Furthermore, tight junction formations and adherens junction formations were confirmed with immunofluorescence analysis (Fig. 1f). This *in vitro* BBB model simulated BBB *in vivo*, allowing the detailed study of cell–cell interactions *in vitro*. Moreover, transendothelial electrical resistance (TEER) was used to measure the formation of tight junctions by brain microvessel endothelial cells, which indicates the integrity of BBB. The TEER of this model was sufficiently high to serve as a BBB *in vivo* (Fig. 1g)^16^,^17^.

### Inhibition of EV secretion suppresses invasiveness through BBB

**Figure 2:**
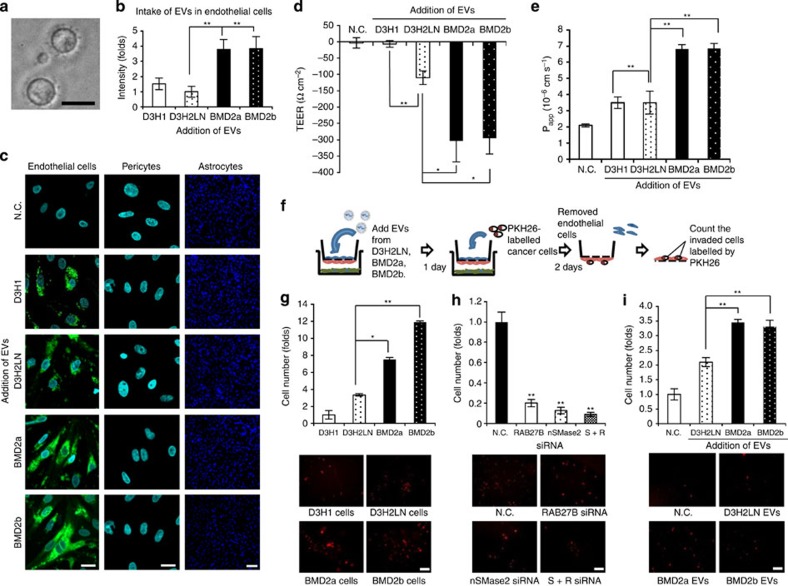
EVs from brain metastatic cancer cells were incorporated into endothelial cells, regulating the invasion through BBB of cancer cells. (**a**) Phase-contrast electron microscopy was used to visualize resuspended EVs pellets. Scale bar, 100 nm. (**b**) The intensity of PKH67-labelled EVs was measured by ImageJ. Error bars represent s.d., Student’s *t*-test, *n*=3. (**P<0.01). (**c**) EVs isolated from cancer cells were labelled using PKH67 and added to the upper chamber. Representative pictures of endothelial cells, pericytes and astrocytes are shown. Negative control (N.C.), EVs from MDA-MB-231-luc-D3H2LN (D3H2LN), EVs from BMD2a and EVs from BMD2b are shown. Scale bar, 20 μm. Bar in the panel of astrocytes represents 100 μm. (**d**) The value of the TEER was monitored before (day 4) and after (day 5) the addition of EVs isolated from each cell line. EVs isolated from brain metastatic cancer cells were incubated in the *in vitro* BBB model for 24 h. Error bars represent s.d., Student’s *t*-test, *n*=3. (**P<0.01, *P<0.05). (**e**) Assessment of BBB permeability determined by NaF (molecular weight=376.27). EVs from MDA-MB-231-luc-D3H1 (D3H1), D3H2LN, BMD2a or BMD2b cells and N.C. were added to the *in vitro* BBB model. After 24 h, NaF was added. NaF that had passed through BBB was measured by a fluorometer. Error bars represent s.d., Student’s *t*-test, *n*=3. (**P<0.01). (**f**) PKH26-labelled cancer cells (2 × 10^4^ cells) were added to the *in vitro* BBB model. After a 48-h incubation, endothelial cells were removed, and the invading cells were counted using a fluorescence microscope. (**g**) *In vitro* BBB transmigration activity of D3H1, D3H2LN, BMD2a or BMD2b cells. The number of transmigrated cells relative to the D3H1 cell lines is plotted. Error bars represent s.d., Student’s *t*-test, *n*=3. (*P<0.05, **P<0.01). (**h**) *In vitro* BBB transmigration activity of the BMD2a treated with control siRNA (N.C.), RAB27B, nSMase2 and both siRNAs (S+R). The number of transmigrated cells relative to the control cells is plotted. Error bars represent s.d., Student’s *t*-test, *n*=3. (**P<0.01). (**i**) The number of transmigrated D3H1 cells relative to the control cells is plotted. Error bars represent s.d., Student’s *t*-test, *n*=3 (**P<0.01). Data are representative of at least three independent experiments each. (Fig. b,c,d,e,g,h,i).

Because BBB consists of three different types of cells, knowing which cells incorporated EVs from cancer cells was essential for determining the precise mechanism of BBB destruction by EVs. The EVs from these cell lines were typical in size (~100 nm) and expressed conventional exosomal markers, such as CD63 and CD9 but not Cytochrome *C* (Fig. 2a, Supplementary Fig. 1a,b), which is a mitochondrial intermembrane-space protein known to be lacking in EVs^18^. The levels of EV secretion did not differ among the brain metastatic and D3H2LN cell lines (Supplementary Fig. 1c). To this end, we added PKH67-labelled EVs isolated from BMD2a, BMD2b and D3H2LN cells to the *in vitro* BBB model. As shown in Fig. 2b,c, EVs from all the cancer cells were incorporated into endothelial cells but not into pericytes or astrocytes. Interestingly, we observed higher fluorescent intensity in the endothelial cells with BMD cell-derived EVs, suggesting that BMD cell-derived EVs undergo tropism in brain endothelial cells (Fig. 2b). To determine whether the EVs from brain metastatic cancer cells functionally affected the destruction of BBB, we added D3H2LN cell-, BMD2a cell- and BMD2b cell-derived EVs to the *in vitro* BBB model, and the TEER of each well was measured. As shown in Fig. 2d and Supplementary Fig. 1d, the TEER was significantly reduced in wells containing the BMD2a- and BMD2b cell-derived EVs compared with those containing the D3H2LN cell-derived EVs (*P*<0.05) as a control or low-metastatic breast cancer cell line MDA-MB-231-luc-D3H1 (D3H1) cell-derived EVs (*P*<0.01). Furthermore, we tested the permeability of BBB using sodium fluorescein (NaF). Although the molecular weight of NaF is low (*M*_W_: 376.27), it cannot significantly permeate the intact BBB^19^. Therefore, NaF is used to assess the permeability of BBB by measuring its concentration with a fluorescence monochromator. NaF showed a high apparent permeability coefficient (Papp) in wells containing BMD2a cell- (*P*<0.01) and BMD2b cell- (*P*<0.01) derived EVs as compared with those containing EVs derived from the D3H2LN cell line or the D3H1 cell line (Fig. 2e). Breast cancer cells are known to attach to microvessel endothelial cells to invade BBB (extravasation) during metastasis to the brain. Brain metastatic cancer cells are also considered to be highly invasive^1^. Matrigel invasion chamber assays were used to confirm the pathological implications of the metastatic potential of established cell lines. As shown in Supplementary Fig. 2a,b, BMD2a and BMD2b cells were more invasive than the D3H2LN cell line or a low-metastatic cell line D3H1. However, the morphology of BMD2a and BMD2b cells did not differ from that of the D3H2LN cell line (Supplementary Fig. 2c). To confirm the extravasation of BMD2a and BMD2b cells into the brain parenchyma side of this model, D3H1, D3H2LN, BMD2a and BMD2b cells were labelled for PKH26 to distinguish them *in vitro* and were added to the upper chamber of the *in vitro* BBB model. Cells that had infiltrated the abluminal side were counted after 2 days (Fig. 2f). As shown in Fig. 2g, more BMD2a and BMD2b cells had extravasated compared with cells from the D3H2LN cell line or the D3H1 cell line, indicating that our established cell lines had a high potential for extravasation through BBB into the brain parenchymal side. To clarify the contribution of EVs to the extravasation of brain metastatic cells, we assessed the extravasation of BMD2a cells in the *in vitro* BBB model after the EV secretion in these cell lines was inhibited by siRNAs against EV secretion-related proteins, such as neutral sphingomyelinase 2 (nSMase2) and RAB27B (Supplementary Fig. 2d,e)^14^,^20^. PKH26-labelled cells treated with control siRNA could still pass through BBB to the abluminal side, but cells in which the production of EVs was inhibited (Supplementary Fig. 2f) were not found on the abluminal side, even when those cells had the ability to pass through BBB (Fig. 2h). In addition, inhibiting the production of EVs only slightly reduced the invasiveness of these cell lines, as assessed by a Matrigel invasion assay (Supplementary Fig. 2g). This finding indicates that extravasation was not only a result of the cells’ invasive potential. Furthermore, to investigate whether the addition of EVs is sufficient to allow the extravasation of cancer cells to the parenchymal side of the brain, we examined the extravasation of low-metastatic D3H1 cells after the addition of D3H2LN cell- and BMD2a cell- and BMD2b cell-derived EVs into the *in vitro* BBB model. The EVs isolated from BMD2a, BMD2b and D3H2LN cells were added to each well and were incubated for 24 h before the addition of PKH26-labelled D3H1 cells. After 2 days, we counted the number of infiltrated cells. As shown in Fig. 2i, D3H1 cells could not invade BBB without the addition of EVs. However, infiltration of D3H1 cells to the abluminal side was significantly increased by the addition of BMD2a cell- and BMD2b cell-derived EVs. By contrast, the addition of D3H2LN cell-derived EVs did not efficiently promote the infiltration of low-metastatic D3H1 cells through BBB compared with BMD2a- and BMD2b cell-derived EVs. Taken together, these results suggest that the secretion of EVs by breast cancer cells affected the extravasation of metastatic cancer cells to the brain parenchyma side across BBB.

### Cancer cell-derived EVs promote brain metastasis *in vivo*

**Figure 3:**
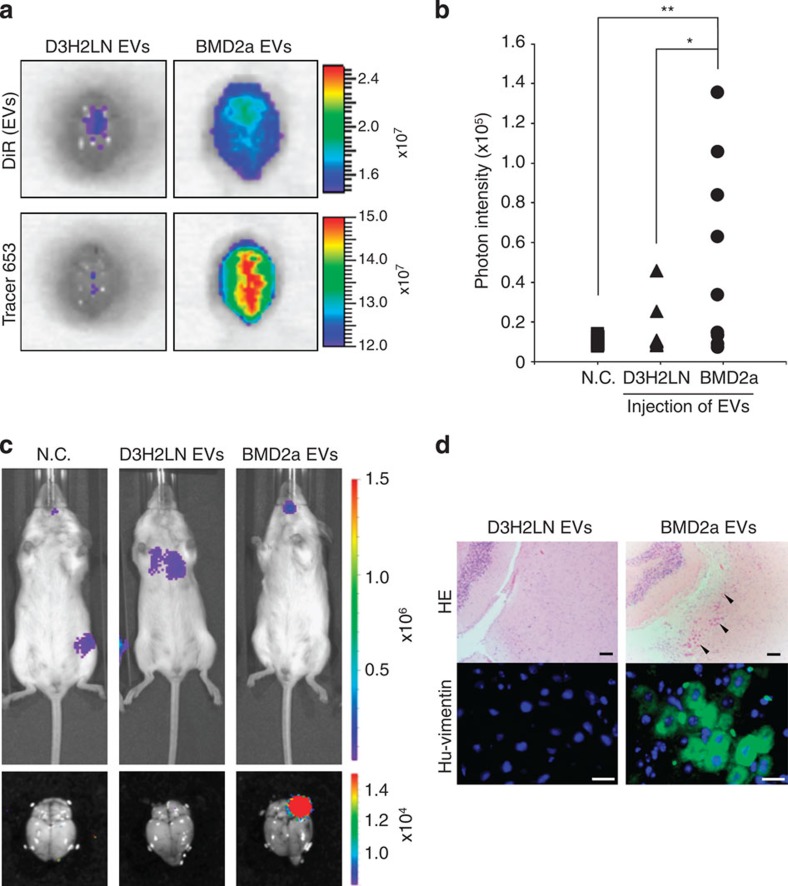
Cancer-derived EVs promoted the brain metastasis of breast cancer cells. (**a**) Fluorescence image of a mouse brain injected D3H2LN or BMD2a cell-derived EVs. The upper image represents the uptake DiR-labelled EVs of a mouse brain. The lower image represents the permeability of a mouse brain. D3H2LN cell-derived EVs were used as a control. This experiment was repeated twice. (**b**) Distribution of photon intensity in the brain, quantified by ImageJ analysis. *P* values were determined by Mann–Whitney one-tailed testing. Negative control (N.C.); *n*=9, D3H2LN; *n*=9, BMD2a; *n*=9. (*P<0.05, **P<0.01). (**c**) Bioluminescence image of D3H2LN and BMD2a cell-derived EVs and N.C.-injected mice. The upper image represents the bioluminescence whole-body image of mice. The lower image represents the bioluminescence image of a mouse brain with cancer cell metastasis. (**d**) Representative image of HE-stained sections from a mouse brain cerebral cortex (upper panels). The arrowhead shows the cancer cells. Scale bar, 100 μm. The lower panel shows a representative immunofluorescence image of anti-human vimentin (Hu-vimentin). Scale bar, 20 μm. Data are representative of at least three independent experiments each (Fig. d).

To evaluate the effect of cancer-derived EVs on the brains of mice, *in vivo* permeability assay was performed. Purified EVs derived from D3H2LN and BMD2a cells were labelled with a XenoLight DiR fluorescent dye before injection into the tail vein of mice. D3H2LN cell-derived EVs were used as a control. After 6 h, Tracer-653 probe, an *in vivo* tracer dye for monitoring BBB disruption, was injected into the mice. The upper panels of Fig. 3a show the intake of EVs and the lower panels show the permeability of brain blood vessels. As shown in the upper panels of Fig. 3a, BMD2a cell-derived EVs were more incorporated within the brains of mice than were D3H2LN cell-derived EVs. As shown in the lower panels of Fig. 3a, BMD2a cell-derived EV-treated mice showed greater permeability of brain blood vessels as compared with D3H2LN cell-derived EV-treated mice *in vivo*.

Furthermore, to clarify the contribution of the EVs to brain metastasis *in vivo*, D3H2LN cell- or BMD2a cell-derived EVs were injected in the tail vein of mice. After 24 h, D3H2LN cells were injected in the left cardiac ventricle of mice; the mice were then observed after 18 days (Supplementary Fig. 3). Importantly, mice treated with the BMD2a cell-derived EVs (brain metastases: 5 out of 9) had more brain metastases as compared with mice treated with D3H2LN cell-derived EVs (brain metastases: 1 out of 9; Table 1). Moreover, mice injected with BMD2a cell-derived EVs had a greater metastatic burden in the brain (*P*<0.05, as measured by luciferase intensity, Fig. 3b) as compared with the mice treated with D3H2LN cell-derived EVs (Fig. 3b). However, negative control-injected mice were not observed to exhibit brain metastasis (Table 1). In other words, mice treated with BMD2a cell-derived EVs had a higher rate of metastasis in the brain as compared with the mice treated with the negative control or mice treated with EVs derived from the D3H2LN cells (Table 1 and Fig. 3c, lower panels). Brain metastasis was confirmed by HE staining and immunofluorescence staining against human vimentin (Fig. 3d). In mice treated with BMD2a cell-derived EVs, but not with D3H2LN cell-derived EVs, the presence of cancer cells in the brain was confirmed by HE staining or immunofluorescence staining (Fig. 3d). These results show that BMD2a cell-derived EVs promoted brain metastasis by increasing the permeability of brain blood vessels, which resulted in BBB breakdown.

### Disruption of intercellular junctions causes BBB breakdown

**Figure 4:**
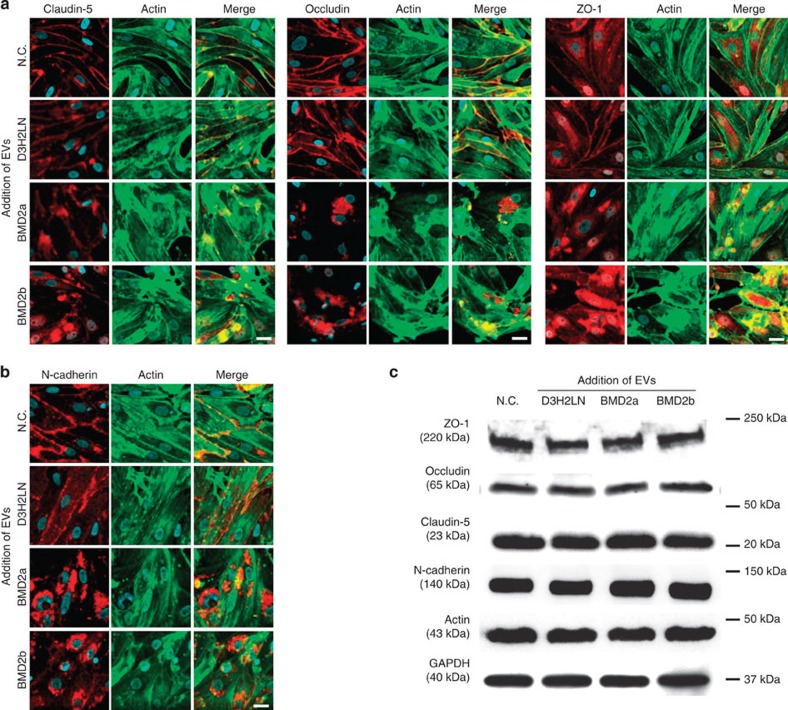
EVs from brain metastatic cancer cells promoted BBB breakdown. (**a**) Co-immunofluorescence of tight junction proteins (Claudin-5, Occludin and ZO-1) (red) and actin filaments (green) after the addition of EVs from D3H2LN, BMD2a or BMD2b cells. Scale bar, 20 μm. (**b**) Co-immunofluorescence of N-cadherin (red) and actin filaments (green) after the addition of EVs from D3H2LN, BMD2a or BMD2b cells. Scale bar, 20 μm. (**c**) Western blot analysis of tight junction proteins, N-cadherin, Actin and GAPDH. Proteins from endothelial cells treated with negative control (N.C.) or EVs. This experiment was repeated twice. Data are representative of at least three independent experiments each (Fig. a,b).

Tight junctions are known to regulate the low permeability of BBB and are formed by specific proteins in endothelial cells, such as Claudin-5, Occludin and ZO-1. On the other hand, N-cadherin, a calcium-dependent cell–cell adhesion glycoprotein composed of five extracellular cadherin repeats that allow it to mediate strong cell–cell adhesion, is mostly expressed on the apical and basal membranes. Tight junction proteins and N-cadherin regulate cell polarity through their intimate association with the actin cytoskeletal network. From these aspects, we hypothesized that the breakdown of BBB was caused by the dysregulation of these molecules. To prove this hypothesis, endothelial cells in the *in vitro* BBB model were co-immunofluorescently stained against Claudin-5, Occludin, ZO-1 and N-cadherin with actin filaments after the addition of EVs isolated from brain metastatic cancer cell lines or the D3H2LN cell line. Tight junction proteins were localized to the surface of the cell membrane in endothelial cells treated with PBS or EVs from D3H2LN cells; however, tight junction proteins and N-cadherin localized to the cytoplasm in cells that were treated with BMD2a cell- and BMD2b cell-derived EVs (Fig. 4a,b). Of note, we found that the expression of tight junction proteins, N-cadherin and actin was not affected by the addition of BMD2a cell- and BMD2b cell-derived EVs to brain blood vessel endothelial cells (Fig. 4c). These results strongly suggested that cancer-derived EVs changed the localization of tight junction proteins, N-cadherin and actin filaments, but did not affect the expression levels of these proteins.

### EV-contained miR-181c diminishes actin filament organization

**Figure 5:**
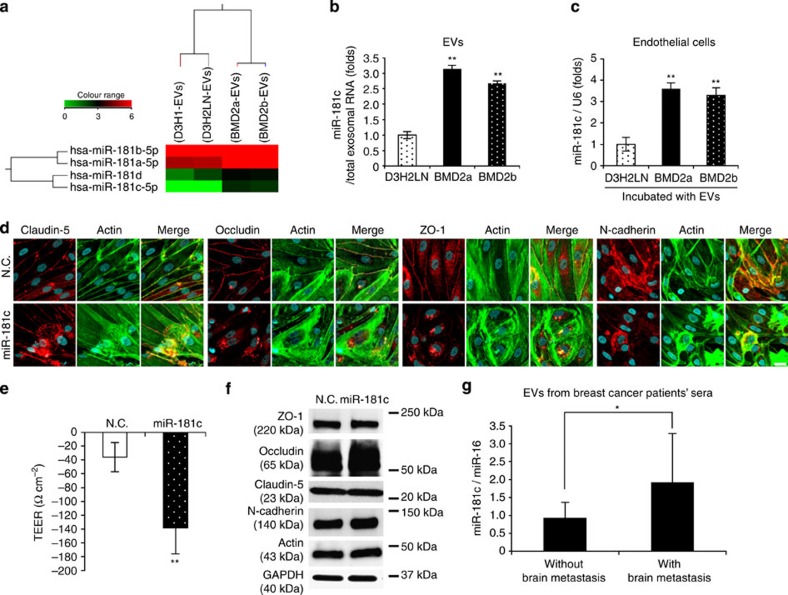
miR-181c played a role in BBB breakdown and upregulation in brain metastasis patients’ sera. (**a**) Heat map showing expression levels of the miR-181c in cancer-derived EVs. (**b**) Amount of miR-181c in EVs isolated from D3H2LN, BMD2a and BMD2b cells. Error bars represent s.d., Student’s *t*-test, *n*=3. **P<0.01 as compared with EVs from D3H2LN cells. (**c**) Endothelial cells were incubated with EVs isolated from D3H2LN, BMD2a or BMD2b cells for 24 h. RNA was isolated from the endothelial cells 24 h after the addition of EVs, and the expression of miR-181c in the endothelial cells was analysed by qRT-PCR. Each bar represents the mean s.d., Student’s *t*-test, *n*=3. **P<0.01 as compared with endothelial cells treated with EVs from D3H2LN cells. (**d**) Co-immunostaining of Claudin-5, Occludin, ZO-1, N-cadherin (red) and actin filaments (green) in endothelial cells after the addition of EVs from D3H2LN, BMD2a or BMD2b cells. Scale bar, 20 μm. These proteins localized to the cytoplasm in miR-181c-transfected cells. (**e**) The TEER value was monitored before (day 4) and after (day 5) the transfection of miR-181c or control siRNA. Transfected miR-181c or N.C. siRNA was incubated in the *in vitro* BBB model for 24 h. Error bars represent s.d., Student’s *t*-test, *n*=3. (**P<0.01). (**f**) Western blot analysis of tight junction proteins, N-cadherin, actin and GAPDH. Proteins were from endothelial cells transfected with N.C. siRNA or miR-181c. This experiment was repeated twice. (**g**) Amount of miR-181c in EVs isolated from patients’ sera. Non-brain metastasis: *n*=46, brain metastasis: *n*=10 (*P<0.05). Associations between the miR-181c expression levels of serum from breast cancer patients were assessed by Student's *t*-test. Data are representative of at least three independent experiments each (Fig. b,c,d,e).

To further investigate the molecular mechanisms by which EVs mediated the abnormal localization of tight junction proteins and adherence junction protein, we performed proteomic and miRNA microarray analysis of EVs isolated from BMD2a, BMD2b and D3H2LN cells. Although the proteomic analysis did not reveal EV protein candidates, we found that miR-181c was significantly upregulated in BMD2a cell- and BMD2b cell-derived EVs as compared with those derived from D3H2LN cells (Fig. 5a,b and Supplementary Fig. 4a). On the contrary, there was not significant expression of cellular miR-181c in D3H2LN, BMD2a or BMD2b cells (Supplementary Fig. 4b). Moreover, we assessed the expression of miR-181c in endothelial cells after the addition of EVs from breast cancer cells and found that its expression was significantly increased by the addition of EVs isolated from BMD2a and BMD2b cells (Fig. 5c). To assess the direct effect of miR-181c on endothelial cells, synthetic miR-181c was transfected into endothelial cells from the *in vitro* BBB model. As shown in Fig. 5d, tight junction proteins, N-cadherin and actin localized to the cytoplasm in miR-181c-transfected cells, although localization of these molecules can be formed on membranes in negative control-treated cells. Indeed, transfection of miR-181c significantly downregulated the value of TEER in the *in vitro* BBB model (Fig. 5e). Furthermore, we found that the expression of tight junction proteins, N-cadherin and actin was not affected by the transfection of miR-181c into brain blood vessel endothelial cells (Fig. 5f).

It is possible that miR-181c in EVs from the brain-metastasized cancer cells could be found in sera from breast cancer patients who have metastases in the brain due to the leakage of circulating EVs from brain metastatic cancer. To clarify this hypothesis, we analysed the expression abundance of miR-181c in sera from breast cancer patients (*n*=56). As shown in Fig. 5g, miR-181c in EVs collected from brain metastasis patients’ serum were significantly higher compared with non-brain metastasis patients (brain metastasis; *n*=10, non-brain metastasis; *n*=46, *P*<0.05, *t-test*). As shown in Supplementary Fig. 4c, the serum level of miR-181c was significantly higher in the sera from brain metastasis patients (stage IV; *n*=10) as compared with the sera from non-brain metastasis patients (stage III; *n*=13; *P*<0.05, stage IV; *n*=6; *P*<0.05, Mann–Whitney *U-test*). Interestingly, in stage IV patients, the miR-181c level was higher in the sera from brain metastasis patients (*P*<0.05). This result emphasizes that secreting miR-181c is related to brain metastasis of breast cancer patients. Taken together, these results suggest that EVs from brain metastatic cancer cells induce the abnormal localization of tight junction proteins by transferring miR-181c into endothelial cells, which results in the destruction of the cell–cell contact.

### PDPK1-regulated actin localization is important to BBB

**Figure 6:**
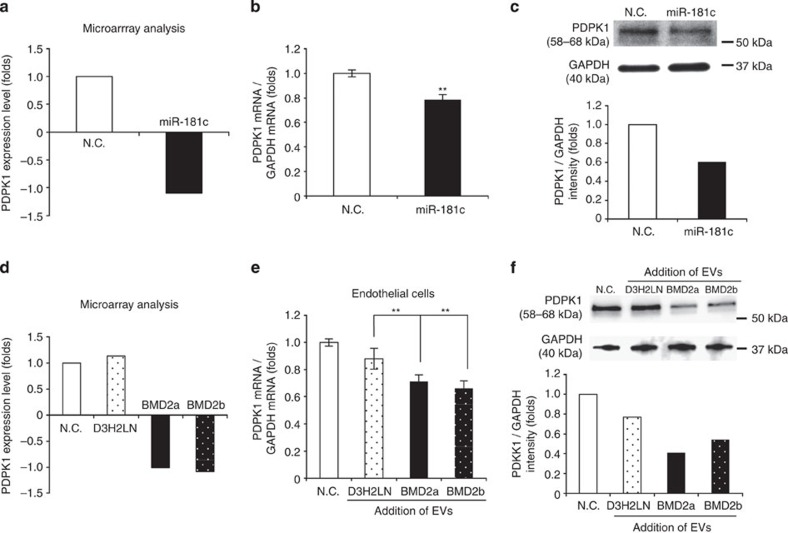
miR-181c regulates PDPK1 expression in brain endothelial cells. (**a**) Microarray analysis showing the expression levels of PDPK1 in brain endothelial cells after transfection of miR-181c. The data are represented as log_2_ value. (**b**) Expression level of PDPK1 mRNA in brain endothelial cells after the transfection of miR-181c. Error bars represent s.d., Student’s *t*-test, *n*=3. (**P<0.01). (**c**) Western blot analysis of PDPK1 and GAPDH. Proteins were from brain endothelial cells transfected with miR-181c. The lower panel shows the intensity of PDPK1 obtained from the transfection of N.C. siRNA or miR-181c. This experiment was repeated twice. (**d**) Microarray analysis showing the expression levels of PDPK1 in brain endothelial cells after EV treatment. (**e**) Expression level of PDPK1 mRNA in brain endothelial cells after the addition of EVs from D3H2LN, BMD2a or BMD2b cells. Error bars represent s.d., Student’s *t*-test, *n*=3 (**P<0.01). (**f**) Western blot analysis of PDPK1 and GAPDH. Proteins were from endothelial cells treated with EVs from D3H2LN, BMD2a or BMD2b cells. The lower panel shows the intensity of PDPK1 obtained from D3H2LN, BMD2a or BMD2b cells. This experiment was repeated twice. Data are representative of at least three independent experiments each (Fig. b,e).

To understand in more detail the molecular mechanism of miR-181c in EV-mediated BBB destruction, we decided to identify the target gene of miR-181c in endothelial cells. We performed global gene expression analysis in endothelial cells after the transfection of negative control or miR-181c (Supplementary Fig. 5a and Fig. 6a) and or after the addition of EVs from BMD2a, BMD2b or D3H2LN cells (Supplementary Fig. 5b and Fig. 6d). We found that overexpression of miR-181c in brain endothelial cells exhibited the downregulation of PDPK1 as compared with the control cells by mRNA and protein expression (Fig. 6b,c). Furthermore, we also found that the expression of PDPK1 was downregulated in BMD2a cell- or BMD2b cell-derived EV-treated endothelial cells as compared with that in D3H2LN cells by mRNA and protein expression (Fig. 6d–f). These results suggest that miR-181c in EVs suppressed the expression of PDPK1 in brain endothelial cells.

**Figure 7:**
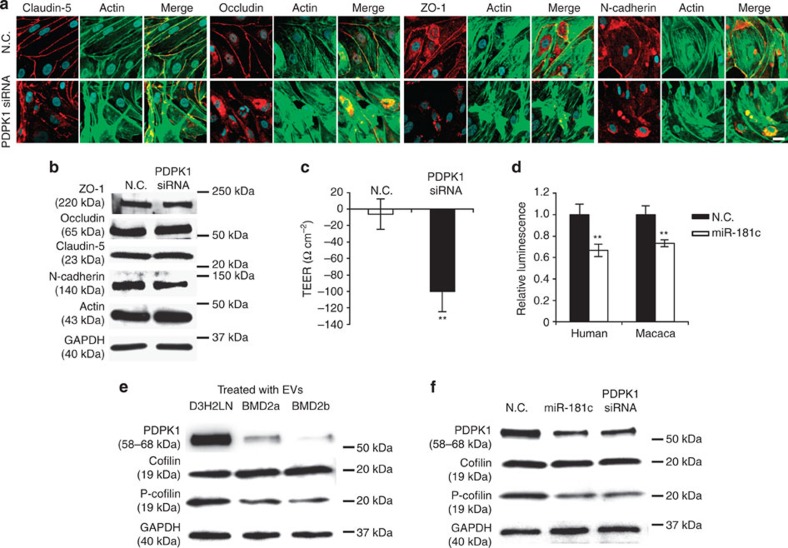
The target of miR-181c, PDPK1, in endothelial cells regulates the localization of tight junction proteins, N-cadherin and actin. (**a**) Co-immunofluorescence of tight junction proteins (Claudin-5, Occludin and ZO-1), N-cadherin (red) and actin filament (green) after the addition of EVs from D3H2LN, BMD2a or BMD2b cells. Scale bar. 20 μm. (**b**) Western blot analysis of tight junction proteins, N-cadherin, actin and GAPDH. Proteins were from brain endothelial cells treated with PDPK1 siRNA. This experiment was repeated twice. (**c**) The TEER value was monitored before (day 4) and after (day 5) the transfection of PDPK1 siRNA or negative control. Error bars represent s.d., Student’s *t*-test, *n*=3 (*P<0.01). (**d**) Luciferase activities measured by cotransfecting miR-181c and the PDPK1 luciferase reporters. Error bars represent s.d., Student’s *t*-test, *n*=6 (**P<0.01). (**e**) Western blot analysis of PDPK1, cofilin, phospho-cofilin (P-cofilin) and GAPDH. Proteins were from brain endothelial cells treated with EVs from D3H2LN, BMD2a or BMD2b cells. This experiment was repeated twice. (**f**) Western blot analysis of PDPK1, phospho-cofilin (P-cofilin), cofilin and GAPDH. Proteins were from brain endothelial cells treated with miR-181c or PDPK1 siRNA. This experiment was repeated twice. Data are representative of at least three independent experiments each (Fig. a,c,d).

Next, we analysed the effect of PDPK1 in the localization of tight junction-related proteins, N-cadherin and actin filaments in brain endothelial cells after the treatment with PDPK1 siRNA. We showed that the expression of PDPK1 protein was downregulated by the transfection of PDPK1 siRNA (Supplementary Fig. 5c,d). As shown in Fig. 7a, we observed the localization of tight junction proteins and N-cadherin at the cellular membrane in control siRNA-treated cells; however, these localizations were dysregulated in PDPK1 siRNA-treated cells. Localization of tight junction proteins, N-cadherin and actin was found in cytoplasm, whose results were the same as those of BMD2a cell- or BMD2b cell-derived EV-treated cells or miR-181c-transfected cells. Interestingly, actin condensation was observed in the endothelial cells (Figs 4a,b, 5d and ). We confirmed that the expression of tight junction proteins, N-cadherin and actin proteins expression was not changed with or without PDPK1 siRNA treatment (Fig. 7b). The TEER of the *in vitro* BBB model was significantly downregulated by PDPK1 siRNA treatment (Fig. 7c). Furthermore, 3′-untranslated region (3′UTR) luciferase reporter assay was performed to analyse miR-181c and PDPK1 mRNA. This result showed that 3′UTR of PDPK1 was a direct target of miR-181c (Supplementary Fig. 5e, and Fig. 7d). Previous reports have shown that PDPK1 is an upstream protein of cofilin phosphorylation^21^,^22^. Cofilin is a family of actin-binding proteins, which disassembles actin filaments, that is activated with dephosphorylation. Finally, western blot analysis of phosphocofilin was performed. Phosphorylation of cofilin in BMD2a cell- or BMD2b cell-derived EVs-treated brain endothelial cells was downregulated as compared with D3H2LN cell-derived EVs treatment (Fig. 7e). Furthermore, phosphorylation of cofilin in miR-181c or PDPK1 siRNA-treated brain endothelial cells was downregulated as compared with negative control siRNA treatment (Fig. 7f). Taken together, these results suggest that miR-181c in EVs modulates the actin dynamics through the downregulation of PDPK1 in brain endothelial cells (Supplementary Fig. 6).

## Discussion

Circulating cancer cells have been shown to adhere to the brain blood vessel endothelium and, subsequently, to infiltrate the brain parenchyma^7^. During this event, cancer cells secrete humoral factors to induce the destruction of BBB and allow extravasation. Previous reports have shown that vascular endothelial growth factor expression of cancer cells was necessary but insufficient for the production of brain metastasis^8^. Bos *et al.*^13^ showed that the expression of ST6GALNAC5 in breast cancer cells enhances their adhesion to brain endothelial cells and their passage through the BBB. One of the key features of brain metastasis is the destruction of BBB^5^. In this article, we have clearly shown that brain metastatic cancer cell-derived EVs trigger BBB destruction to promote the extravasation of cancer cells through BBB. BBB is composed of tight junction proteins, such as Claudin-5, Occludin and ZO-1. The primary cytoskeletal protein, actin, has known binding sites on all ZO proteins and on claudins and Occludin. Actin filaments serve both structural and dynamic roles in the cell^1^. Previous findings have shown that PDPK1 protein has a reduced F-actin to G-actin ratio^23^ and is a critical regulator of actin polymerization^24^. Active cofilin is an actin-binding protein that severs filaments. Phosphorylation of cofilin through PDPK1 is thought to inactivate cofilin in a spatial manner in which local activation occurs at the cell membrane. We have demonstrated that the actin dynamic in BBB was regulated with cancer-derived EVs in brain metastases. In this article, we have shown that PDPK1 was downregulated by miR-181c *in vitro* BBB model. Decreased PDPK1 in brain endothelial cells resulted in the breakdown of BBB by activated cofilin. Many reports have shown that miR-181c is related to malignancies such as hepatocellular carcinoma^25^, basal cell carcinoma^26^ and breast cancer^27^. Our results support the possibility that miR-181c has an important role in malignancy.

Recently, Zhou *et al.*^15^ reported that breast cancer cell-derive EVs contributed to BBB breakdown. Despite the significance of their findings, we should emphasize that our study is novel in the following points. First, our study suggests the possibility of organ tropism in brain metastasis of breast cancer cells. Zhou *et al.* revealed the possibility of the breakdown of the junctions between endothelial cells throughout the body. In contrast, our study focuses the brain-oriented metastasis of breast cancer, by establishing brain-oriented metastatic cell lines. Of note, we observed that EVs derived from these cell lines were more prone to accumulate in the brain than those from the parental cell line (Fig. 3a). Second, the mechanism described in the present study provides a novel miRNA-associated mechanism for EV-mediated BBB breakdown. Zhou *et al.*’s work suggests that miR-105 suppressed the ZO-1 expression in endothelial cells, and the resulting loss of cell–cell adhesion lead to the promotion of metastasis. However, considering the capacity of EVs to harbour a variety of miRNAs, it seems difficult to conclude that a single EV-miRNA can explain the entire mechanism of BBB breakdown. In this regard, we revealed another novel mechanism in which miR-181c contributed to actin degradation through the suppression of cofilin. Our findings, along with those by Zhou *et al.*, will help to understand the EV-mediated BBB breakdown.

Despite our observation that EVs accumulated preferentially in the brain, we have not yet identified the molecules responsible for this organ tropism. Molecular analysis of EV membrane components will be of great importance to elucidate the organ tropism of metastatic cancer-derived EVs. In addition, the degree of contribution of EV-delivered miR-181c to BBB breakdown in the brain is not yet clear. Nevertheless, we have detected an increased level of circulating miR-181c in breast cancer patients with brain metastasis, suggesting an important role of secretory miR-181c in brain metastasis.

EVs as therapeutic targets might aid the prevention of BBB destruction in brain metastatic cancer and other diseases. The phenomenon of BBB destruction is not limited to brain metastases. BBB destruction is also known to be involved in diabetes, stroke, trauma, Alzheimer’s disease, brain tumours and malaria^28^ that lead to disorders in brain function^29^,^30^. On the other hand, BBB destruction mechanism by cancer-derived EVs might be a useful drug-delivery system for brain through BBB. This BBB breakdown mechanism includes the possibility of developing to drug-delivery system using EVs. Furthermore, EVs from cells contain multiple molecules, such as mRNAs, miRNAs and proteins as well as membrane-associated molecules. These molecules might affect recipient cells in multiple ways and might contribute to target organ tropism of circulating EVs.

In conclusion, this study provides new insights into the mechanisms of brain metastatic cancer.

## Methods

### Isolation of brain metastatic cells

A cell suspension containing 2 × 10^5^ MDA-MB-231-luc-D3H2LN breast cancer cells in a volume of 100 μl was injected into the left cardiac ventricle of anaesthetised 7-week-old C.B-17 Icr-scid scid female mice. Tumour development was monitored by weekly bioluminescence imaging using the IVIS Spectrum (Caliper Life Science, Hopkinton, MA). Brain metastatic lesions were confirmed by histological analysis after necropsy. Brain lesions were localized by *ex vivo* bioluminescence imaging and resected under sterile conditions. Half of the tissue was fixed with 4% paraformaldehyde and processed for histological analysis. The other half was minced and placed in RPMI1640 culture medium (Gibco) containing antibiotic–antimycotic agents and 10% fetal bovine serum (FBS). The cells were briefly centrifuged, resuspended in 0.025% trypsin–EDTA (Gibco), and incubated for an additional 10 min at 37 °C. The cells were resuspended in a culture medium containing 50 μg ml^−1^ Zeocin (Gibco) and were allowed to grow to confluence on a 10-cm dish. BMD2a and BMD2b cells were sorted for further propagation in culture or inoculation in mice. All animal work followed a protocol approved by the NCC Institutional Animal Care and Use Committee (T12-005, T12-005-M01).

### Cell culture

MDA-MB-231-luc-D3H1 (purchased from Xenogen Co., CA), MDA-MB-231-luc-D3H2LN (purchased from Xenogen Co., CA), BMD2a and BMD2b (established from MDA-MB-231-luc-D3H2LN) breast cancer cell lines were cultured in RPMI1640 medium and supplemented with 10% heat-inactivated FBS (Invitrogen) and antibiotic–antimycotic agents at 37 °C in 5% CO_2_.

### Transwell invasion assay

For invasion assays, 2 × 10^4^ cells were plated in the top chamber with a Matrigel-coated membrane (24-well insert, BD Biosciences, NJ, USA). Cancer cells were plated in RPMI1640 medium without FBS and RPMI1640 medium supplemented with 10% FBS (for all other cells) was used as a chemoattractant in the lower chamber. The cells were incubated for 24 h, and cells that did not migrate or invade through the pores were removed using a cotton swab. Cells on the lower surface of the membrane were stained with the Diff-Quick Staining Set (Sysmex, Hyogo, Japan) and counted. All assays were performed in triplicate. The data are expressed as the invasion percentage through the Matrigel matrix and membrane relative to the migration through the control membrane, according to the manufacturer’s instructions.

### Preparation of conditioned medium and EVs

Before the collection of cultured medium, MDA-MB-231-luc-D3H1, MDA-MB-231-luc-D3H2LN, BMD2a and BMD2b cells were washed three times with Advanced RPMI containing antibiotic–antimycotic agents and 2 mM L-glutamine (medium A), and the medium was switched to fresh medium A. After incubation for 2 days, the medium was collected and centrifuged at 2000*g* for 10 min at 4 °C. To thoroughly remove cellular debris, the supernatant was filtered with a 0.22-μm Stericup (Millipore, MA, USA). To prepare the EVs, the conditioned medium was ultracentrifuged at 110,000*g* for 70 min at 4 °C. The pellets were washed with 11 ml of PBS and resuspended in PBS after ultracentrifugation. The fraction containing the EVs was measured for its protein content using the Micro BCA protein assay kit (Thermo Scientific, MA, USA).

### PKH67-labelled EV transfer

Purified EVs derived from MDA-MB-231-luc-D3H2LN, BMD2a and BMD2b cells were labelled with a PKH67 green fluorescent labelling kit (Sigma-Aldrich, MO, USA). EVs were incubated with 2 μM of PKH67 for 5 min, washed five times using a 100-kDa filter (Microcon YM-100, Millipore) to remove excess dye and incubated for 24 h in the *in vitro* BBB model at 37 °C in 5% CO_2_.

### *In vitro* BBB model

To investigate the function of EVs, the BBB kit (MBT24-H) was used as an *in vitro* blood–brain barrier model (PharmaCo-Cell Co. Ltd, Nagasaki, Japan, http://www.pharmacocell.co.jp/). The TEER value exceeded 150 Ω cm^−2^, which indicates that this system could be used as an *in vitro* BBB model.

### TEER study

BBB function of the *in vitro* BBB model was quantified by its TEER. The resistance values (Ω) were measured with an ohmmeter (Millicell ERS-2, Millipore). The TEER values were calculated by means of the unit area resistance:

*R*=(*A*—*B*) × 0.33 cm^2^

*R*=TEER(Ω cm^−2^)

*A*=measurement resistance value (Ω)

*B*=blank resistance value (Ω)

### Permeability assay

We used a method modified from a previously described permeability assay^31^. After the addition of 200 μl of NaF (10 μg ml^−1^, Sigma-Aldrich) to the upper chamber, as well as the addition of 900 μl of DPBS-H (Dulbecco’s PBS (Mg^+^, Ca^+^) and 10 mM HEPES, 4.5 mg ml^−1^ D-glucose) to the lower chamber, the plate was incubated with shaking at 37 °C. After 30 min, the DPBS-H of the lower chamber was dispensed into a black plate (*n*=8). These samples were measured with a multi-detection monochrometer microplate reader (485/535 nm, SAFIRE, Tecan) and the apparent permeability coefficient (Papp) was calculated as follows:

Papp=(VA·[C]A)·A^−1^·[C]Luminal ^−1^. *t*^−1^

VA: volume of abluminal chamber (0.9 cm^3^)

A: membrane surface area (0.33 cm^2^)

[C]Luminal: initial luminal tracer concentration (μg ml^−1^)

[C]A: abluminal tracer concentration (μg ml^−1^)

t: time of experiment (_min_)

### *In vivo* permeability assay

Purified EVs derived from MDA-MB-231-luc-D3H2LN and BMD2a cells were labelled with a XenoLight DiR (#125964, Summit Pharmaceuticals International Co.). EVs were incubated with 10 mM of XenoLight DiR for 15 min and washed five times using a 100-kDa filter (Microcon YM-100, Millipore) to remove excess dye. A XenoLight DiR-labelled EV, 5 μg in a volume of 100 μl, was injected into the tail vein of anaesthetised 7-week-old C.B-17 Icr-scid scid mice. After 6 h, 100 μl of Tracer-653 probe (TR-1001, Molecular Targeting Technologies Inc., PA, USA) was injected into the tail vein of mice. After 24 h, mice were refluxed to remove excess dye in the blood and were monitored by fluorescence imaging using the IVIS Spectrum (Caliper Life Science, Hopkinton, MA).

### Transwell invasion assay using the *in vitro* BBB model

Breast cancer cell invasion was assessed in an *in vitro* BBB model (PharmaCo-Cell Co. Ltd, Nagasaki, Japan). Cancer cells were trypsinized for the invasion assay and labelled with PKH26 (Sigma-Aldrich) when using the *in vitro* BBB model. Cancer cells (2 × 10^4^ cells) were plated in Ham’s F-12 (Gibco) medium without serum, and the medium supplied by the *in vitro* BBB model kit containing 10% serum was used as the chemoattractant in the lower chamber. After 48 h, non-invading cells were removed with cotton swabs and the nuclei were stained with Hoechst 33342 (Dojindo, Kumamoto, Japan). The PKH26 fluorescence of invading cells was subsequently counted. All assays were performed in triplicate.

### siRNA transfection

RAB27B, nSMase2, and both siRNA transfections of BMD2a cells were performed using the DharmaFECT transfection reagent (Thermo Scientific) according to the manufacturer’s protocol. A total of 25 nM of siRNA was used for each transfection. After 24 h, the transfected cells were used for each assay. miR-181c (Ambion, ID: MC10181; AACAUUCAACCUGUCGGUGAGU) and PDPK1 (Ambion, ID: S10275; UUUCUCACAGCCUAACCGCT) siRNA transfection were performed using the DharmaFECT transfection reagent. A total of 25 nM siRNA was used for each transfection.

### RNA isolation and detection of miRNA and mRNA

The total RNA from the cultured cells or patient sera, which included the efficient recovery of small RNAs, was isolated using the RNeasy Mini Kit (Qiagen). EVs isolated from patients sera were isolated using Total Exosome Isolation (from serum) (Invitrogen). The expression of mRNA and miRNA was assessed with quantitative reverse transcription PCR (qRT-PCR) as described previously^14^. PCR was carried out in 96-well plates using the 7300 Real-Time PCR System (Applied Biosystems, CA, USA). All reactions were performed in triplicate. All TaqMan MicroRNA assays were purchased from Applied Biosystems. RNU6 was used as an internal control. The expression levels of miR-181c and RNU6 were measured by qRT-PCR using a Universal PCR Master Mix (Applied Biosystems). Gene expression was analysed using TaqMan Gene Expression Assays (Applied Biosystems). The expression of RAB27B, nSMase2, β-actin, PDPK1 and GAPDH was measured by qRT-PCR using a Platinum Quantitative PCR SuperMix (Applied Biosystems). Primers and probes were defined as follows: RAB27B (Assay ID: Hs01072206_m1), nSMase2 (Assay ID: Hs00920354_m1), actin-beta (Assay ID: Hs01060665_g1), PDPK1 (Assay ID: Hs00176884_m1) and GAPDH (Assay ID: Hs02758991_g1). GAPDH was used as an internal control.

### Western blot

Proteins were isolated from cells using M-PER (Thermo Scientific, MA, USA) separated in Mini-PROTEAN TGX Gel (4–12%, Bio-Rad) and electrotransferred onto a PVDF membrane (Millipore). After blocking in Blocking One (Nacalai Tesque, Kyoto, Japan), the membranes were incubated for 1 h at room temperature with primary antibodies, which included anti-CD63 (purified mouse anti-human CD63, H5C6, 1:200, BD), anti-CD9 (ALB6, 1:200, Santa Cruz Biotechnology Inc.), anti-cytochrome C (purified mouse anti-cytochrome C, 7H8.2C12, 1:200, BD), anti-Claudin-5 (Z43.JK, 1:200, Invitrogen), anti-Occludin (ZMD.481, 1:200, Invitrogen), anti-ZO-1 (H-300, 1:100, Santa Cruz Biotechnology), anti-N-cadherin (3B9, 1:500, Invitrogen), anti-PDPK1 (#3062, 1:500, Cell Signaling), anti-GAPDH (6C5, 1:1000, Millipore), anti-Cofilin (D3F9, 1:1000, Cell Signaling), anti-Phospho-Cofilin (Ser3) (#3311, 1:500, Cell Signaling) and anti-N-SMase2 (H-195, 1:200, Santa Cruz Biotechnology Inc.). Secondary antibodies (HRP-linked anti-mouse IgG, NA931 or HRP-linked anti-rabbit IgG, NA934, GE Healthcare) were used at a dilution of 1:2,000. The membrane was then exposed to ImmunoStar LD (Wako, Osaka, Japan). Original scans of the cropped images in the figures are presented in Supplementary Figs 7–13.

### Immunofluorescence

Endothelial cells from the *in vitro* BBB model were fixed with 3% paraformaldehyde in PBS for 10 min at room temperature and treated with PBS containing 0.1% Triton X-100 for 10 min after being washed with PBS containing Mg^2+^ and Ca^2+^ to permeabilize the cells. After fixation, the cells were incubated with 3% BSA in PBS for 1 h to block the nonspecific binding of antibodies. Subsequently, the endothelial cells were incubated with rabbit polyclonal antibodies against Claudin-5 (Z43.JK, Invitrogen, CA, USA), Occludin (ZMD.467, Invitrogen), ZO-1 (ZMD.437, Invitrogen) and N-cadherin (3B9, Invitrogen) at 37 °C for 1 h. After washing with PBS containing Mg^2+^ and Ca^2+^, they were incubated with Alexa Fluor 594-conjugated anti-rabbit IgG (Invitrogen) for 1 h at 37 °C. Actin was stained with ActinGreen 488 ReadyProbes Reagent (R37110, Molecular Probes). The stained cells were then washed in PBS without Mg^2+^ and Ca^2+^ and were mounted in VECTASHIELD Mounting Medium (H-1200, Vector Laboratories, CA, USA) for observation under a confocal microscope (FluoView FV1000, Olympus, Tokyo, Japan).

### PDPK1 3′UTR luciferase reporter assay

The 3′UTR for PDPK1 was PCR amplified from total RNA extracted from brain endothelial cells of the *in vitro* BBB model. PCR primers used to amplify the 3′UTR include forward: 5′-AACTCGAGAATGCTGGCTATTGTTGGCCTC-3′ and reverse: 5′-AAGCGGCCGCAAGATTAAATCACTGACCCAATAG-3′. The PCR products were cloned into a pGEM-T Easy Vector (Promega, WI, USA). Amplified 3′UTR were cloned downstream of the *Renilla* luciferase coding region in the psiCHECK-2 (Promega). Human umbilical vein endothelial cells (HUVEC) cells were seeded in 96-well plates 24 h before transfection. The following day, 100 ng of reporter plasmid along with 100 nM of pre-miR-181c was co-transfected using DharmaFECT Duo transfection reagent (Thermo Scientific). HEK293 cells were cultured at a density of 5 × 10^4^ cells per well in 96-well tissue culture plates overnight. Cells were collected 24 h after transfection and assayed for luciferase activity using EnVision (PerkinElmer, MA, USA). To assess the precursor miRNA’s effect on reporter activity, 100 ng of synthetic precursor miRNA (pre-miR; Ambion, Invitrogen) was co-transfected. All experiments were performed in triplicate.

### Microarrays

*miRNA expression of EVs*. Total RNA was extracted from EVs using QIAzol reagent and miRNeasy Mini Kit (Qiagen). RNA quantity and quality were determined using NanoDrop ND-1000 spectrophotometer (Thermo Fisher Scientific Inc.) and Agilent Bioanalyzer (Agilent Technologies), as recommended. Total RNA was labelled with cyanine 3 (Cy3) using miRNA Complete Labeling and Hyb Kit (Agilent Technologies) following the manufacturer’s instructions. In brief, 100 ng of total RNA was dephosphorylated using Calf Intestinal Alkaline Phosphatase Master Mix incubated at 37 °C for 30 min. Dephosphorylated RNA was denatured with dimethylsulphoxide incubated at 100 °C for 5 min and then immediately transfers to on ice for 2 min. These products were mixed with a Ligation master mix for T4 RNA Ligase and Cy3-pCp (Cy3-Cytidine biphosphate) and incubated at 16 °C for 2 h. Labelled RNA was dried using vacuum concentrator at 55 °C for 1.5 h. Cy3-pCp-labelled RNA was hybridized on Agilent SurePrint G3 Human miRNA 8 × 60 K Rel.19 (design ID: 046064). After washing, microarray were scanned using an Agilent DNA microarray scanner. Intensity values of each scanned feature were quantified using Agilent Feature Extraction software version 10.7.3.1, which performs background subtractions. We only used features that were flagged as no errors (Detected flags) and excluded features that were not positive, not significant, not uniform, not above background, saturated and population outliers (not detected flags). This expression analysis was performed with Agilent GeneSpring GX version 12.6.1. There are a total of 2,006 miRNA probes on SurePrint G3 Human miRNA 8 × 60 K Rel.19 (design ID: 046064) without control probes. We applied ≥2-fold change in signal intensity to identify the significant differences of gene expression in this study. Raw and normalized microarray data are available in the Gene Expression Omnibus database (accession numbers GSE63445).

*mRNA expression of cells*. Total RNA was extracted from cultured brain endothelial cells before EVs or miR-181c treatment using the QIAzol reagent and the miRNeasy Mini Kit (Qiagen). RNA quantity and quality were determined using a NanoDrop ND-1000 spectrophotometer (Thermo Fisher Scientific Inc.) and an Agilent Bioanalyzer (Agilent Technologies), as recommended. Total RNA was amplified and labelled with Cy3 using Low Input Quick Amp Labeling Kit, one-colour (Agilent Technologies) following the manufacturer’s instructions. In brief, 100 ng of total RNA was reversed-transcribed to double-strand complementary DNA (cDNA) using a poly dT-T7 promoter primer. Primer, template RNA and quality-control transcripts of known concentration and quality were first denatured at 65 °C for 10 min and incubated for 2 h at 40 °C with 5 × first strand buffer, 0.1 M dithiothreitol, 10 mM deoxynucleotide triphosphate mix and AffinityScript RNase Block Mix. The AffinityScript enzyme was inactivated at 70 °C for 15 min. cDNA products were then used as templates for *in vitro* transcription to generate fluorescent complementary RNA (cRNA). cDNA products were mixed with a transcription master mix in the presence of T7 RNA polymerase and Cy3-labelled CTP (cytidine 5′-triphosphate) and incubated at 40 °C for 2 h. Labelled cRNA was purified using RNeasy Mini Spin Columns (Qiagen) and eluted in 30 ml of nuclease-free water. After amplification and labelling, cRNA quantity and cyanine incorporation were determined using a NanoDrop ND-1000 spectrophotometer and an Agilent Bioanalyzer. For each hybridization, 0.60 μg of Cy3-labelled cRNA was fragmented and hybridized at 65 °C for 17 h to an Agilent Cynomolgus macaque Gene Expression Profiling Array (design ID: 028520). After washing, the microarrays were scanned using an Agilent DNA microarray scanner. Intensity values of each scanned feature were quantified using Agilent Feature Extraction software version 10.7.3.1, which performs background subtractions. We only used features that were flagged as no errors (detected flags) and excluded features that were not positive, not significant, not uniform, not above background, saturated and population outliers (compromised and not detected flags). Normalization was performed with Agilent GeneSpring GX version 12.6.1 (per chip: normalization to 75th percentile shift; per gene: normalization to median of all samples). There are a total of 12,243 probes on Agilent Cynomolgus macaque Gene Expression Profiling Array (design ID: 028520) without control probes. The altered transcripts were quantified using the comparative method. We applied ≥2-fold change in signal intensity to identify the significant differences of gene expression in this study. Raw and normalized microarray data are available in the Gene Expression Omnibus database (accession numbers GSE63447).

### Patient serum samples

Collection and usage of human serum from breast cancer patients (*n*=56) by the National Cancer Center Institute of Japan were approved by the Institutional Review Board (No.2013-111). Serum was aliquoted and stored at −80 °C until used, and freeze-thawing was avoided as much as possible after that. Informed consent was obtained from all the patients.

### Statistical analyses

The data are presented as the mean±s.d. *P* values <0.05 were considered statistically significant by Student’s *t*-test or Mann–Whitney *U-*test.

## Author contributions

N.T. performed the experimental work, analysed the data and wrote the manuscript. N.K. assisted in the writing of the manuscript and provided helpful discussion. K.T. provided the patients’ serum samples. M.O., T.K., Y.Y., H.N. and J.L. provided helpful discussion. The manuscript was finalized by T.O. with the assistance of all of the authors.

## Additional information

**Accession codes.** Raw and normalized microarray data are available in the Gene Expression Omnibus (GEO) database (accession numbers GSE63445, and GSE63447).

**How to cite this article:** Tominaga, N. *et al.* Brain metastatic cancer cells release microRNA-181c-containing EVs capable of destructing the blood–brain barrier *in vitro*. *Nat. Commun.* 6:6716 doi: 10.1038/ncomms7716 (2015).

## Supplementary Material

Supplementary Information Supplementary Figures 1-13

## Figures and Tables

**Table 1:** Brain metastasis rate after EVs treatment.

**Treatment**	**Brain metastasis, % (*****n*****=9)**
N.C.	0 (0%)
D3H2LN EVs	1 (11.1%)
BMD2a EVs	5 (55.6%)

EVs: extracellular vesicles; N.C.: negative control.

D3H2LN: MDA-MB-231-D3H2LN-luc.

The brain metastasis rate after EV treatment. *n*=9 mice per group. D3H2LN cell-derived EVs were used as a control.
