# Supplementary material for: Brain metastatic cancer cells release microRNA-181c-containing extracellular vesicles capable of destructing blood–brain barrier
Source: Nat Commun. 2015 Apr 1;6:6716. doi: 10.1038/ncomms7716 (PMC4396394; doi:10.1038/ncomms7716)
Supplement: Supplementary Information — Supplementary Figures 1-13 [file ncomms7716-s1.pdf]

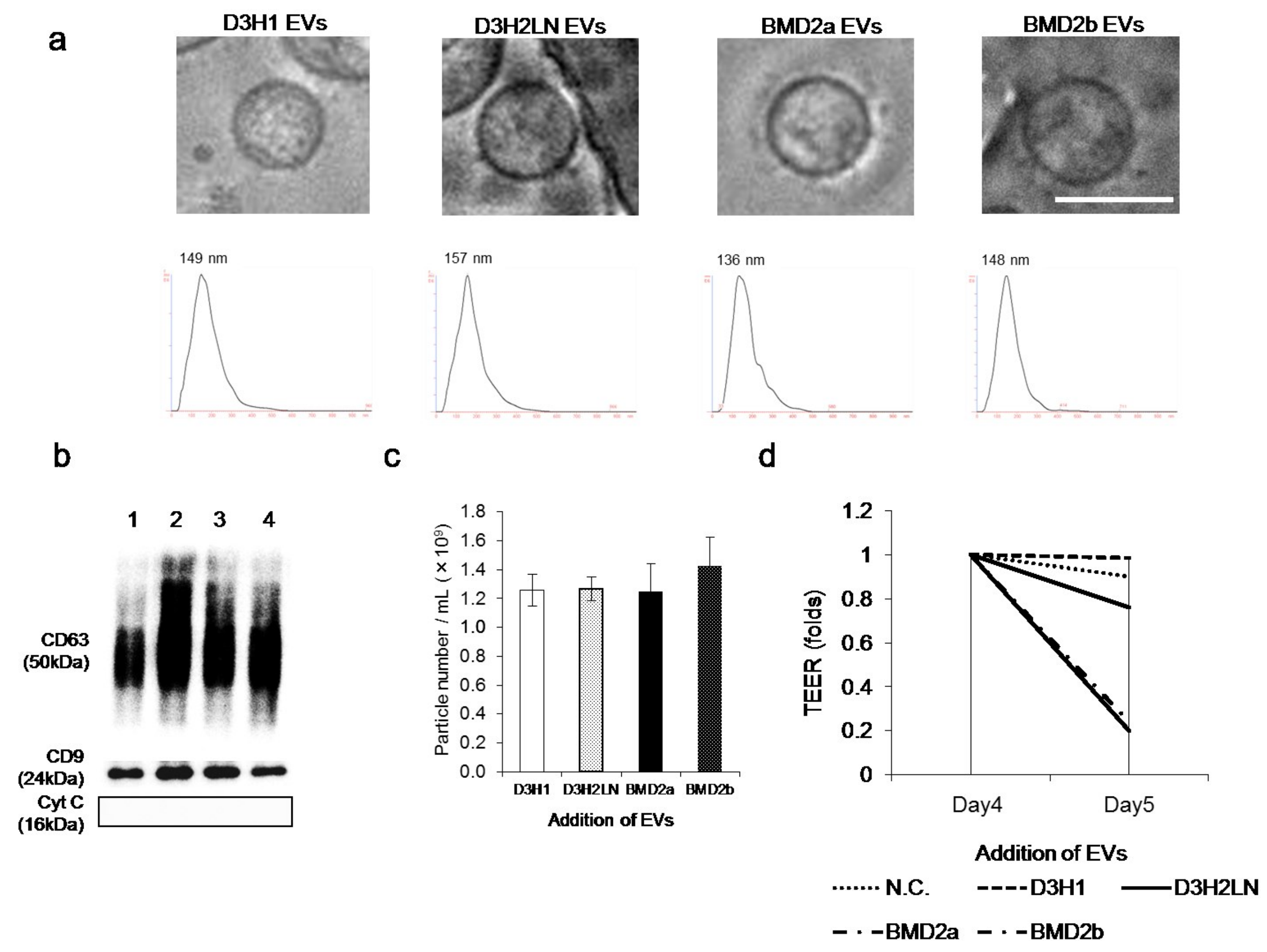

Supplementary Figure 1. Characterization of EVs

(a) Phase-contrast electron microscopy was used to visualize resuspended EV pellets. Scale bar represent 100 nm. The sizes of EVs from MDA-MB-231-D3H1 (D3H1), MDA-MB-231-D3H2LN (D3H2LN), BMD2a, and BMD2b cells were measured by NanoSight. Numbers shown on graphs are the average sizes of all EV types.

(b) The conventional EV markers CD63 (500 ng/lane), CD9 (500 ng/lane), and Cytochrome C (3 μg/lane) were assessed by Western blot analysis. Lane 1: EVs from MDA-MB-231-D3H1; Lane 2: EVs from MDA-MB-231-D3H2LN; Lane 3: EVs from BMD2a; Lane 4: EVs from BMD2b.

(c) Number of EV particles isolated from each cell line as measured by NanoSight.

(d) The change in TEER after the addition of EVs. EVs from D3H1, D3H2LN, BMD2a, or BMD2b cells and negative control (N.C.) were added to the wells on day 4 after thawing of the *in vitro* BBB model. After 24 hrs, the TEER values were measured using an electrical resistance meter.

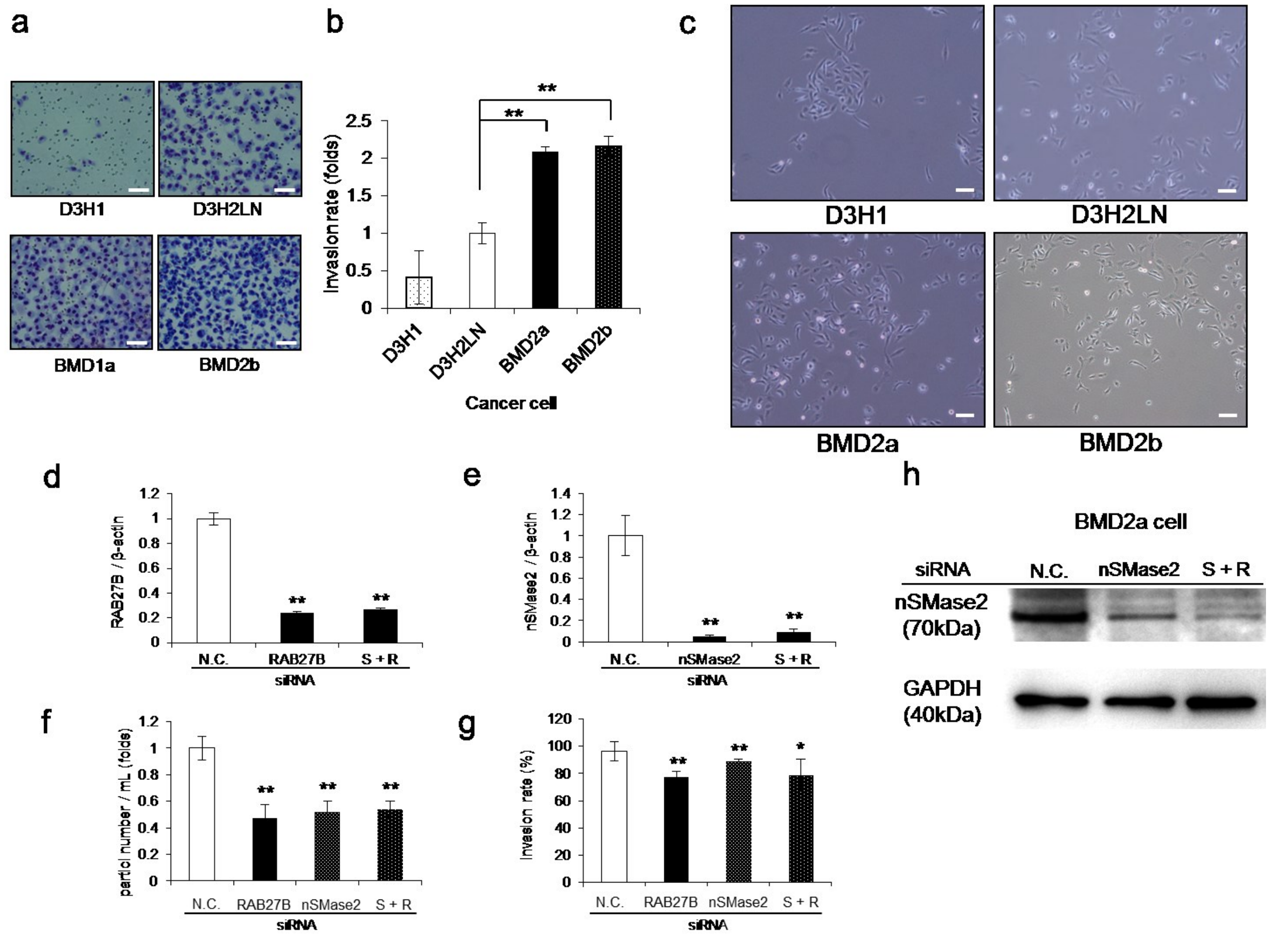

Supplementary Figure 2. Inhibition of EVs secretion decreased invasiveness of cancer cells through BBB.

(a) Representative images of invading cells, including D3H1, D3H2LN, BMD2a, or BMD2b. Bar represents 100 μm.

(b) The invasion rate of BMD2a cells treated with siRNA against EV secretion-related proteins, such as nSMase2 and/or RAB27B and the transfection of control siRNA (N.C.). Inhibiting the production of EVs minimally inhibited the invasiveness of these cell lines, as assessed by a Matrigel™ invasion assay. Error bars represent S.D., n = 3. (** P < 0.01)

(c) Representative cellular morphologies of D3H1, D3H2LN, BMD2a, and BMD2b cells. Bar represents 100 μm.

(d) The expression of RAB27B mRNA in BMD2a cells after the transfection of control siRNA (N.C.), RAB27B siRNA, neutral sphingomyelinase 2 (nSMase2) siRNA, or nSMase2 and RAB27B siRNA (S+R). Error bars represent S.D., n = 3. (** P < 0.01)

(e) The expression of nSMase2 mRNA in BMD2a cells after the transfection of control siRNA (N.C.), nSMase2 siRNA, or nSMase2 siRNA and RAB27B siRNA (S+R). Error bars represent S.D., n = 3. (** P < 0.01)

(f) The number of EVs from siRNA-treated BMD2a cells. BMD2a cells were treated with siRNA against EV secretion-related proteins, such as nSMase2 and/or RAB27B and the transfection of control siRNA (N.C.). Error bars represent S.D., n = 3. (** $P < 0.01$)

(g) *In vitro* BBB transmigration activity of the MD3H1, D3H2LN, BMD2a, and BMD2b cells. The number of transmigrated cells relative to the parental cell lines is plotted. Error bars represent S.D., n = 3. (* $P < 0.05$, ** $P < 0.01$)

(h) Western blot analysis of nSMase2, and GAPDH. Proteins from BMD2a cells treated with N.C. siRNA, nSMase2 siRNA, and nSMase2 + Rab27b siRNA.

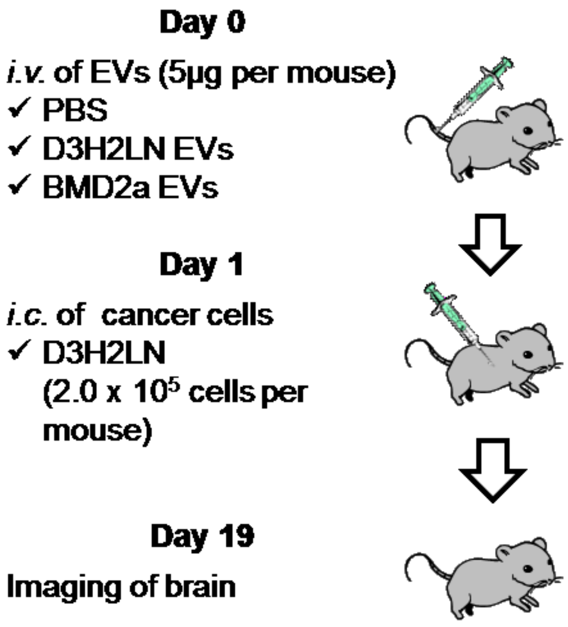

Supplementary Figure 3. Schematic representation of the protocol for the in vivo brain metastasis experiment. PBS (negative control), D3H2LN, and BMD2a-derived EVs (5μg/mouse) were injected intravenously (i.v.) into C.B-17/Icr-scid/scid mice (Day 0). After 24 hrs, MDA-MB-231-D3H2LN breast cancer cell lines (2×10^5 cells) were injected intracardially (i.c.) into C.B-17/Icr-scid/scid mice (Day 1). After 18 days, the brain metastasis of cancer cells was monitored by in vivo imaging system (IVIS). n = 9 mice per group.

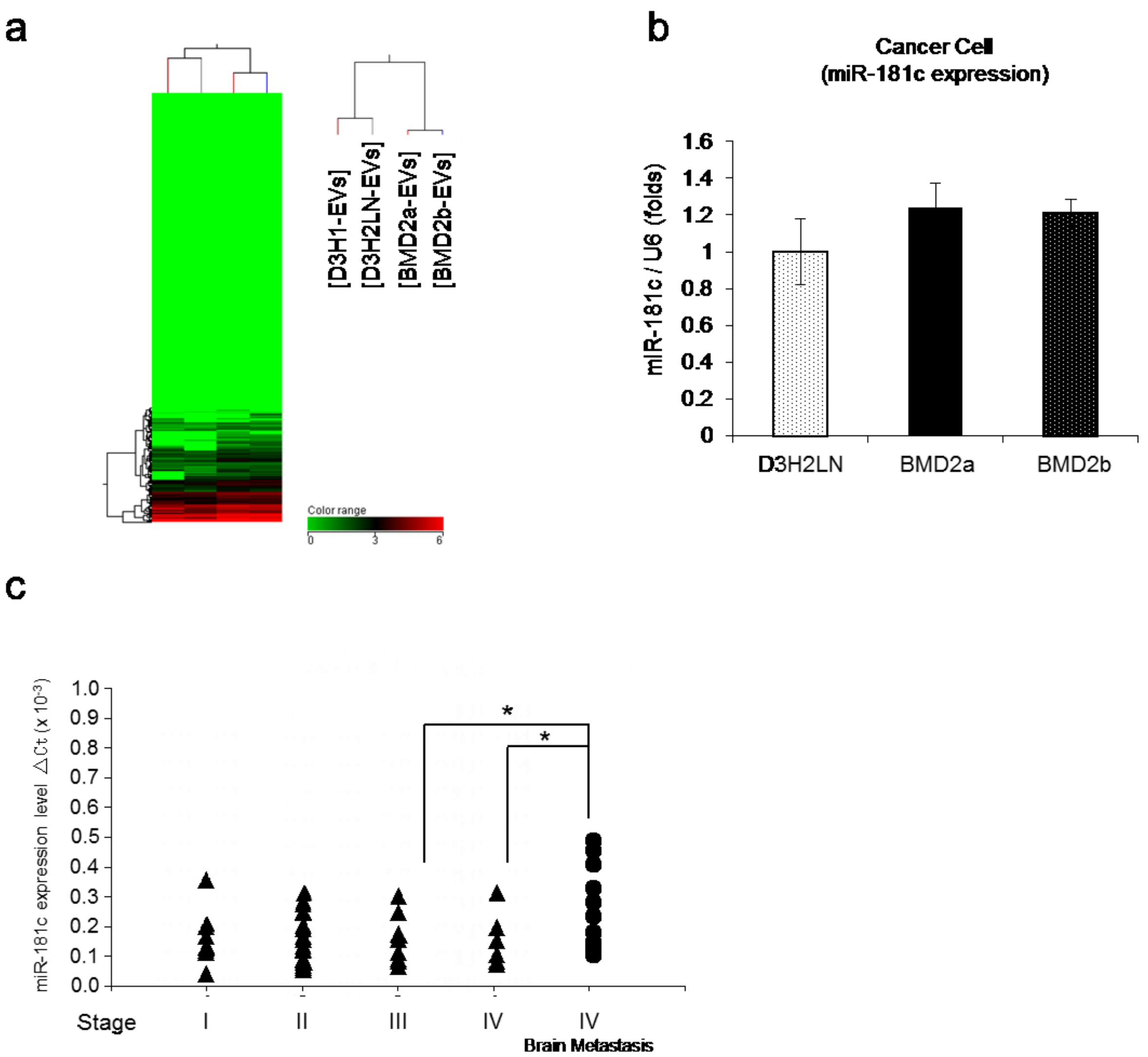

Supplementary Figure 4. miRNA array analysis of EVs

(a) Heat map showing expression levels of the miRNAs in EVs isolated from MDA-MB-231-D3H1 (D3H1), MDA-MB-231-D3H2LN (D3H2LN), BMD2a, or BMD2b cells.

(b) The expression levels of miR-181c in D3H2LN, BMD2a, and BMD2b cells were measured by qRT-PCR. Error bars represent S.D., *n* = 3.

(c) Expression level of miR-181c in total RNA from patients' sera. 1: stage 1 (*n* = 10); 2: stage 2 (*n* = 22); 3: stage 3 (*n* = 9); 4: stage 4 (*n* = 6); 5: brain metastasis patients (*n* = 10). (* $P < 0.05$, ** $P < 0.01$) Associations between the miR-181c expression levels of serum from breast cancer patients were assessed by Mann-Whitney U test.

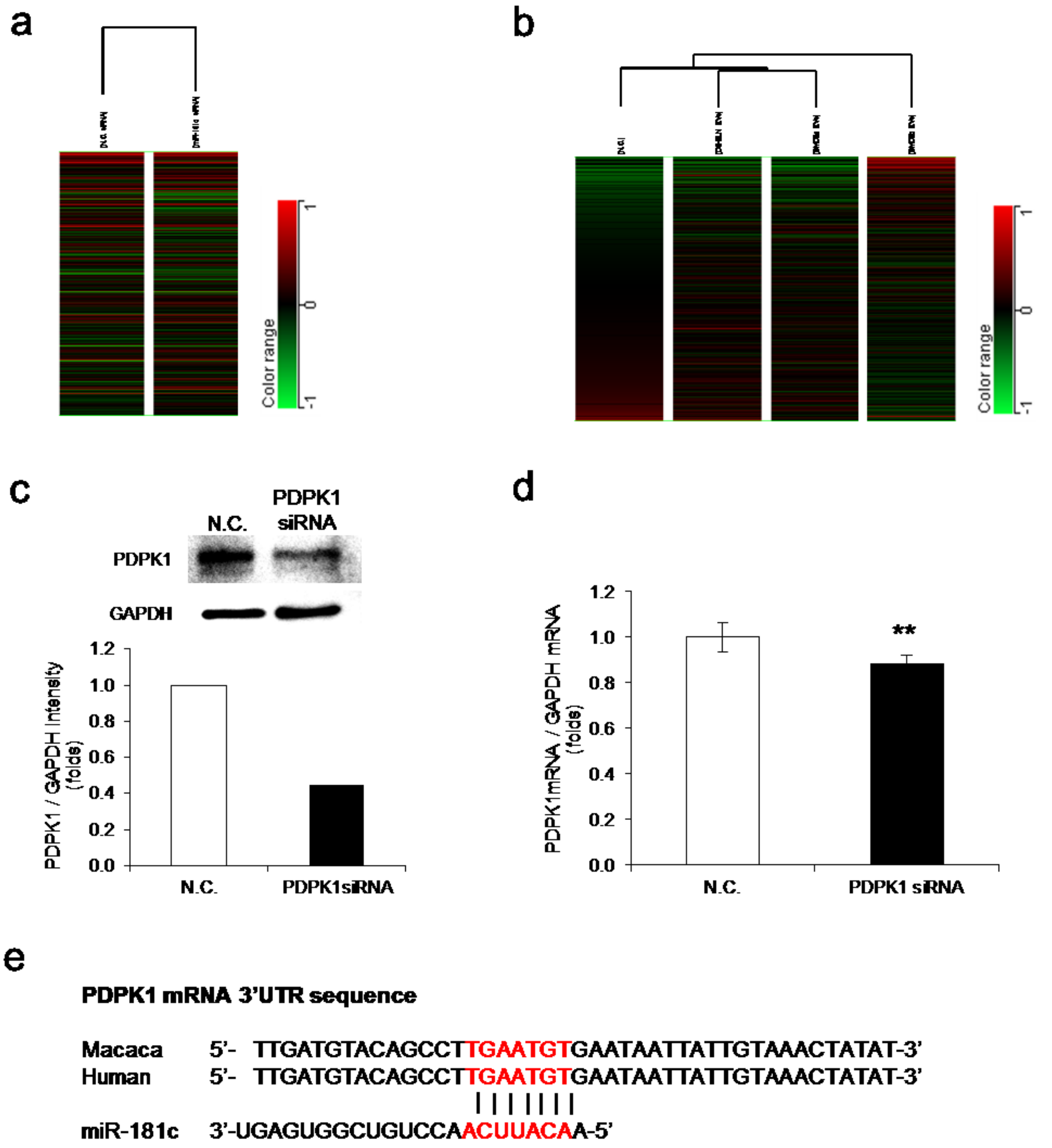

Supplementary Figure 5. miR-181c targeted PDPK1

(a) Heat map showing expression levels of the mRNAs in brain blood vessel endothelial cells transfected N.C. miRNA or miR-181c.

(b) Heat map showing expression levels of the mRNAs in brain blood vessel endothelial cells treated EVs from MDA-MB-231-D3H1 (D3H1), MDA-MB-231-D3H2LN (D3H2LN), BMD2a, or BMD2b cells.

(c) Western blot analysis of PDPK1 and GAPDH. Proteins were from brain endothelial cells treated with PDPK1 siRNA.

(d) The expression levels of PDPK1 in brain endothelial cells after the transfection of PDPK1 siRNA were measured by qRT-PCR. Error bars represent S.D., $n = 3$.

(e) Schematics of the miR-181c binding site within the 3' UTR of the target mRNA.

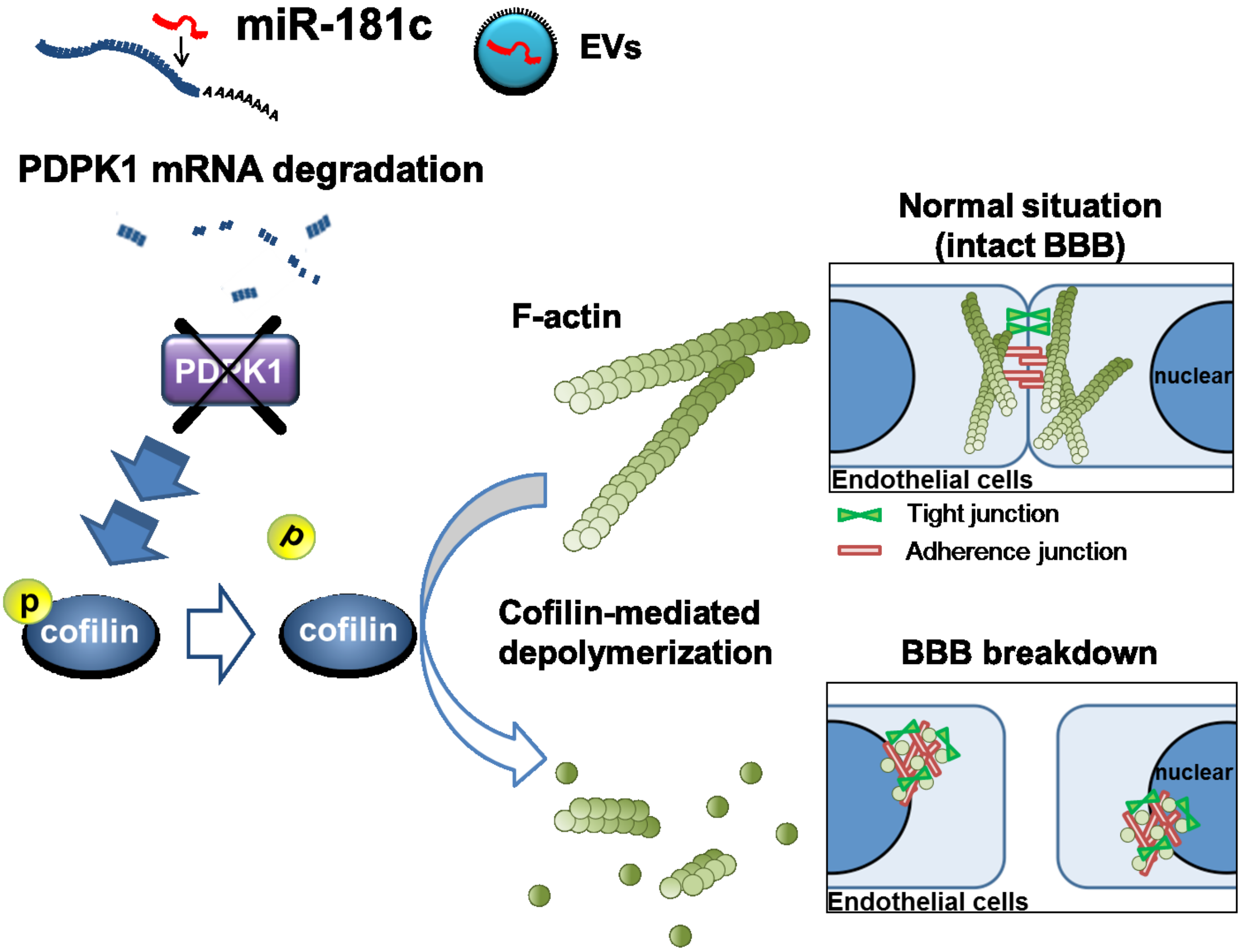

Supplementary Figure 6. Schematic model of BBB breakdown by EVs

Schematic model shows that miR-181c regulates de-phosphorylation of cofilin through PDPK1 expression. PDPK1 was suppressed by extracellular vesicles carrying miR-181c that was incorporated into brain endothelial cells. Activated cofilin is increased because PDPK1 was suppressed. Actin filaments are disassembled with activated cofilin. Tight junction proteins and cadherin were delocalized because actin filaments were destroyed.

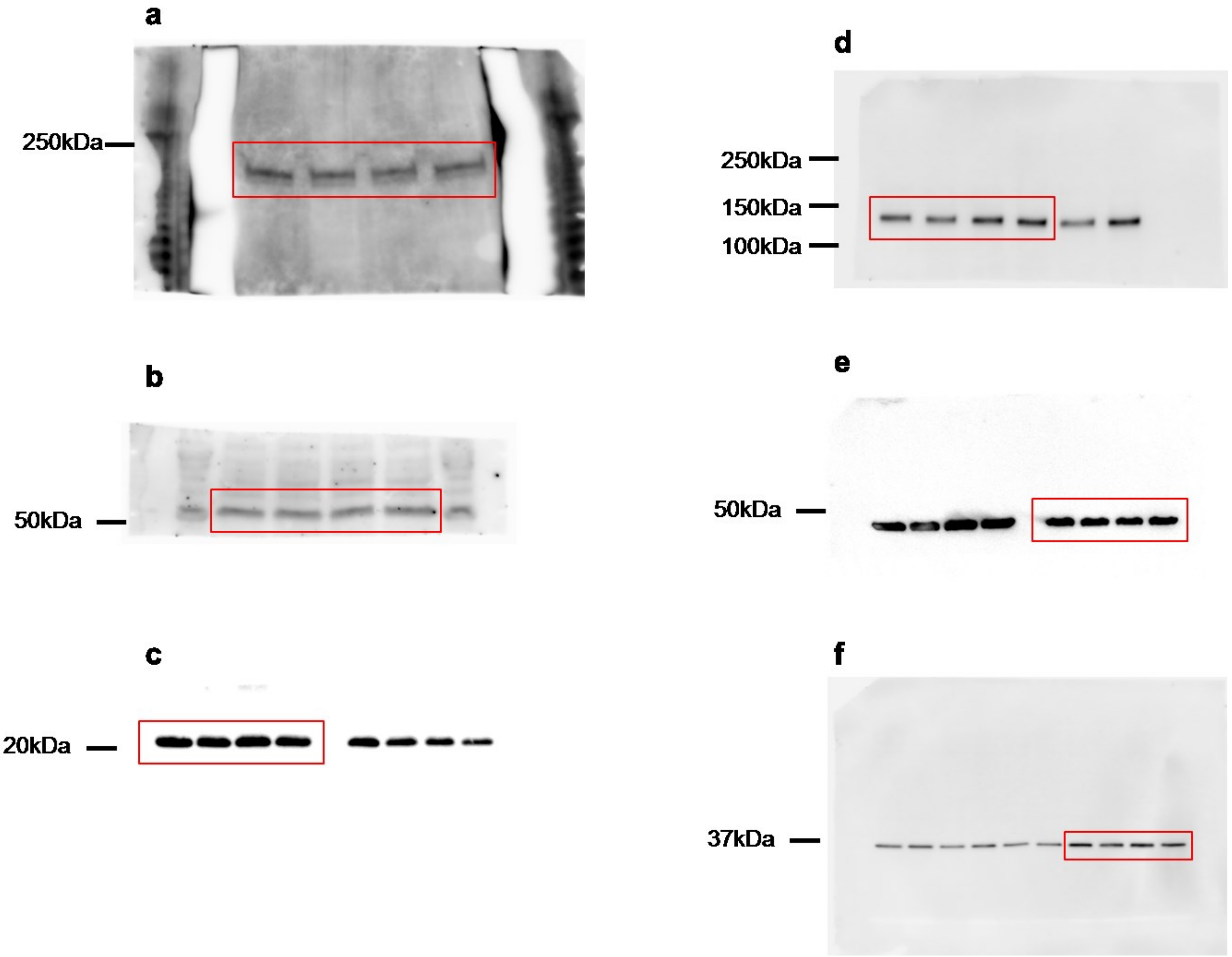

Supplementary Figure 7. Uncropped scans of blots shown in figure 4c.
(a) ZO-1 (b) Occludin (c) Claudin-5 (d) N-cadherin (e) Actin (f) GAPDH

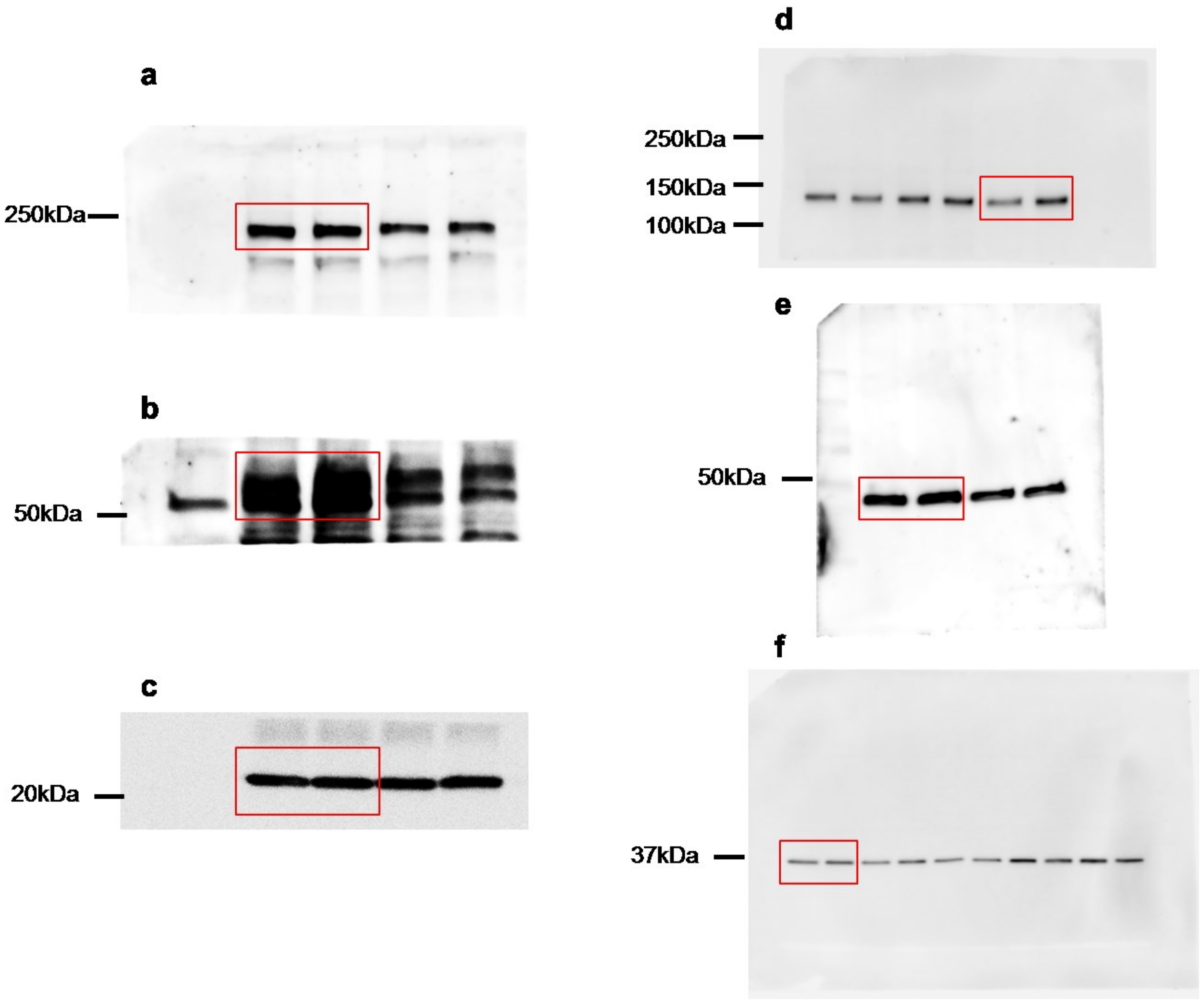

Supplementary Figure 8. Uncropped scans of blots shown in figure 5f.
(a) ZO-1 (b) Occludin (c) Claudin-5 (d) N-cadherin (e) Actin (f) GAPDH

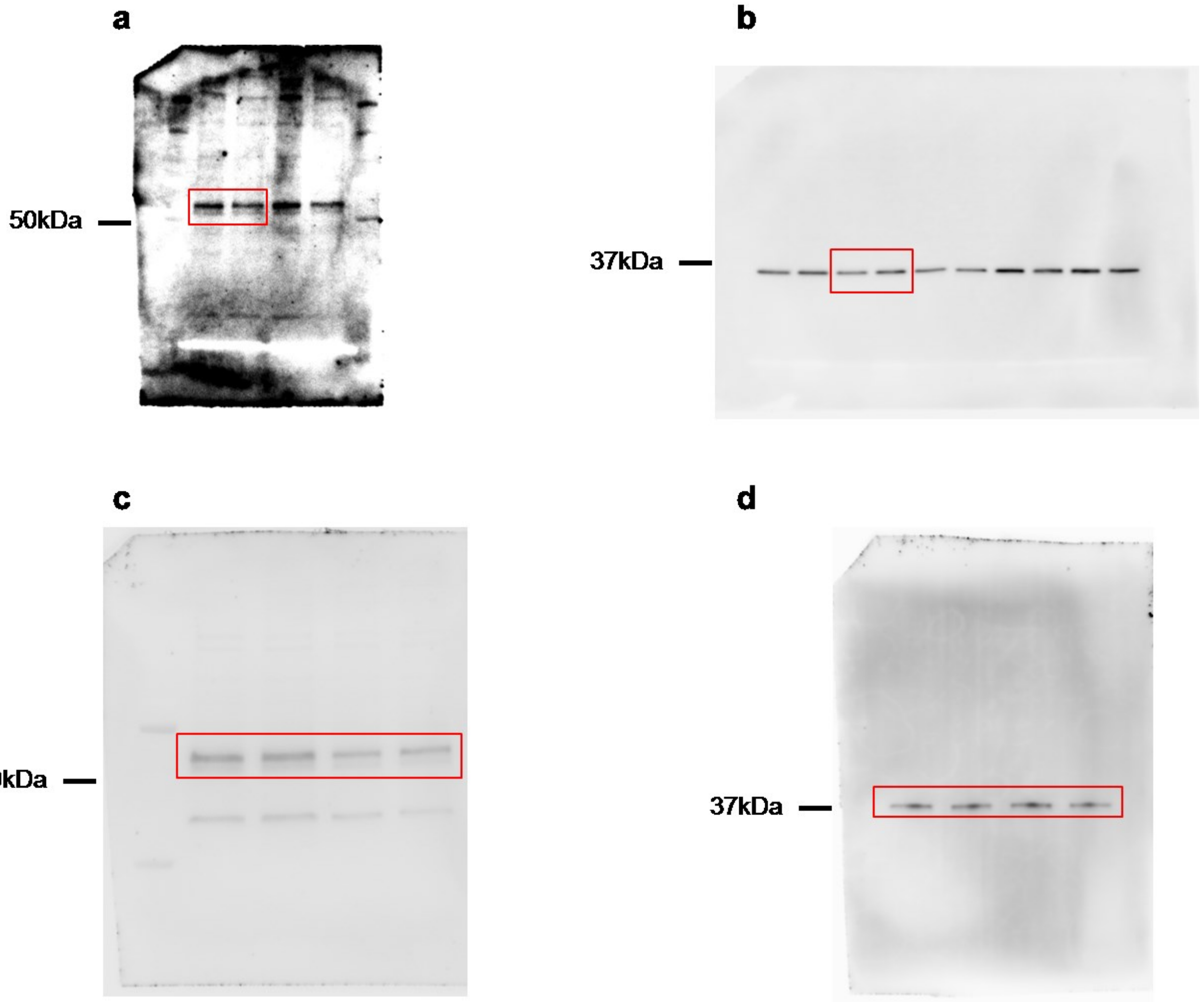

Supplementary Figure 9. Uncropped scans of blots shown in figure 6c and 6f.
(a) Fig.6c, PDPK1 (b) Fig.6c, GAPDH (c) Fig.6f, PDPK1 (d) Fig.6f, GAPDH

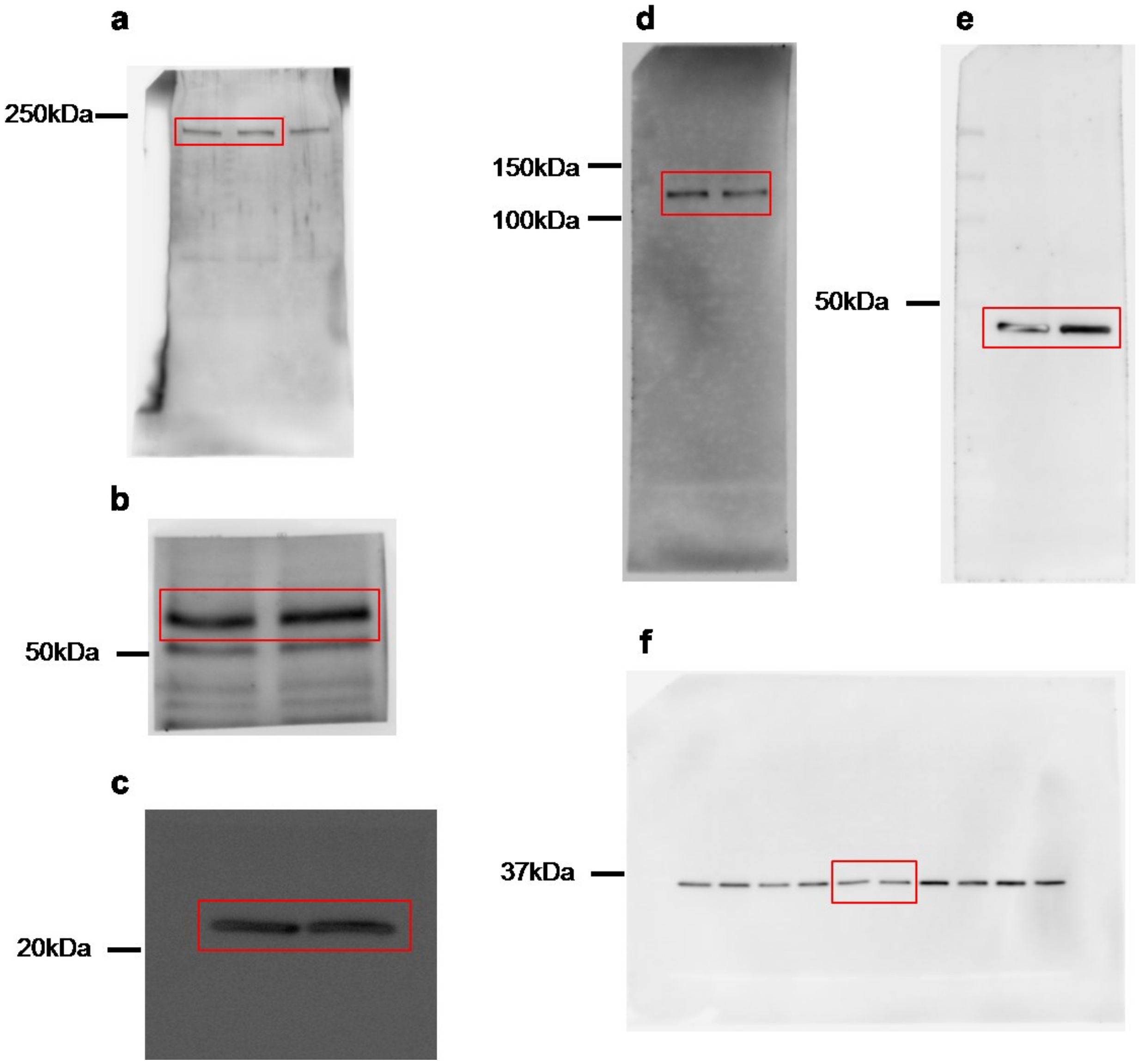

Supplementary Figure 10. Uncropped scans of blots shown in figure 7b.
(a) ZO-1 (b) Occludin (c) Claudin-5 (d) N-cadherin (e) Actin (f) GAPDH

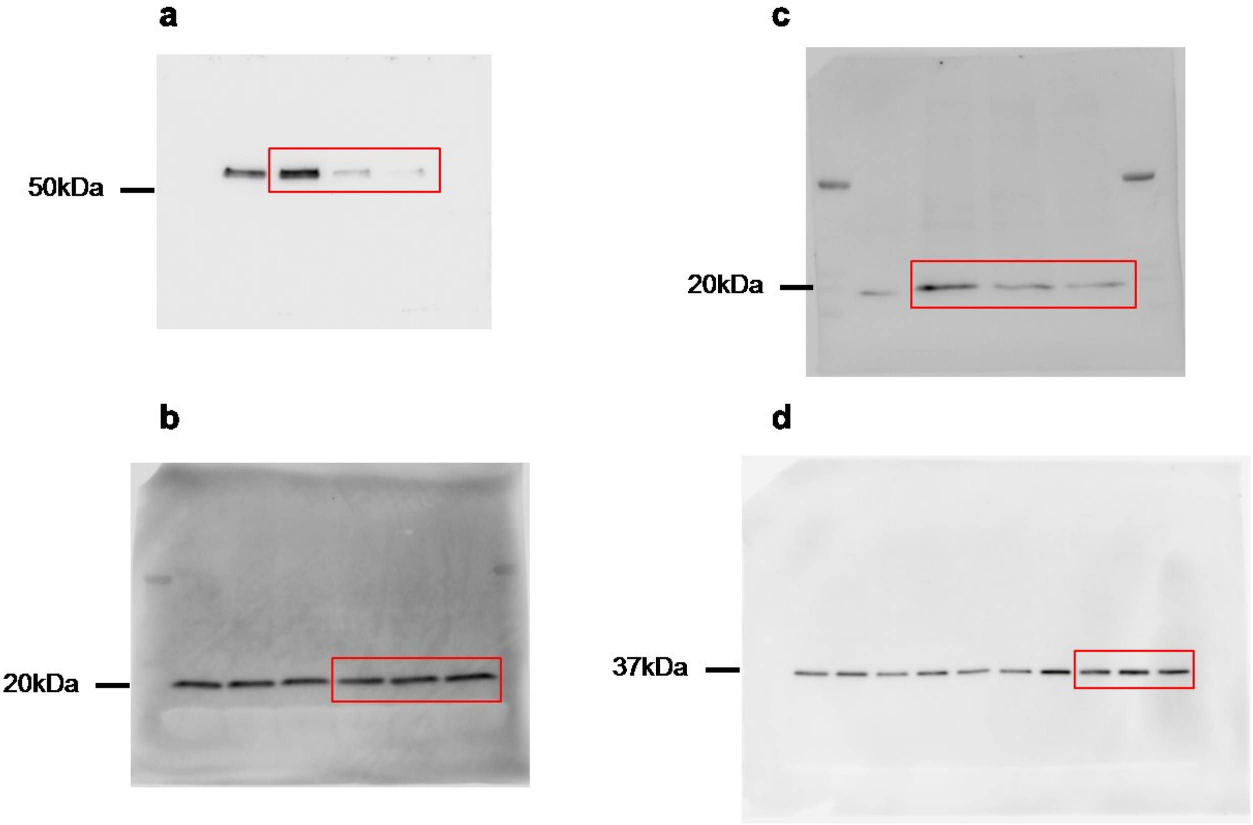

Supplementary Figure 11. Uncropped scans of blots shown in figure 7e.
(a) PDPK1 (b) Cofilin (c) P-Cofilin (d) GAPDH

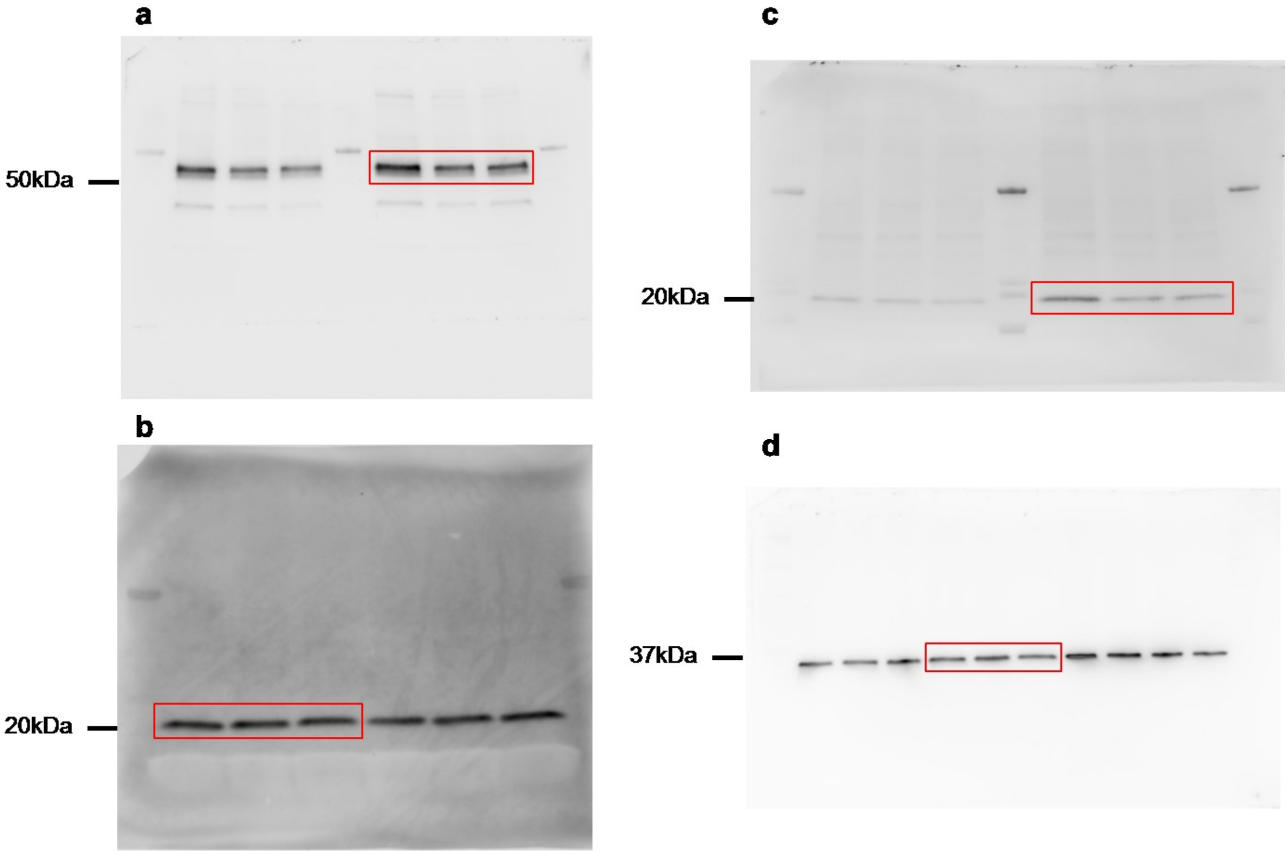

Supplementary Figure 12. Uncropped scans of blots shown in figure 7f.
(a) PDPK1 (b) Cofilin (c) P-Cofilin (d) GAPDH

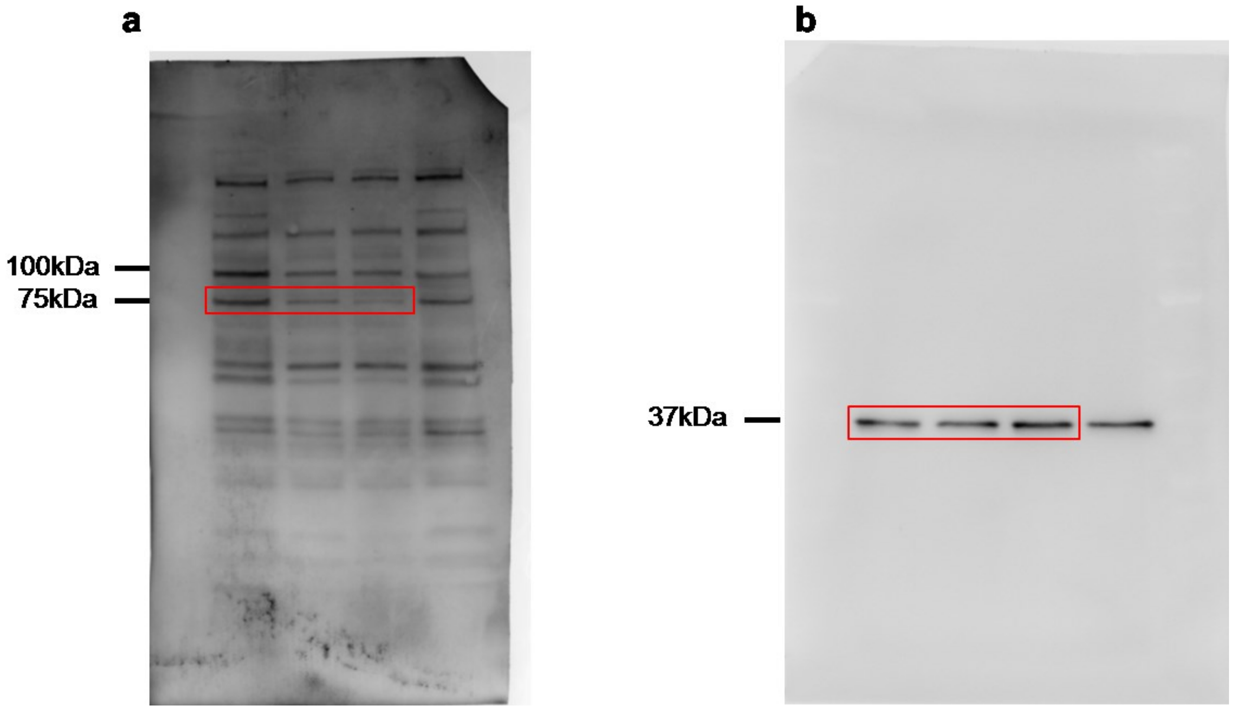

Supplementary Figure 13. Uncropped scans of blots shown in supplementary figure 2h.
(a) nSMase2 (b) GAPDH
